# TLR8 signaling enhances tumor immunity by preventing tumor-induced T-cell senescence

**DOI:** 10.15252/emmm.201403918

**Published:** 2014-09-17

**Authors:** Jian Ye, Chunling Ma, Eddy C Hsueh, Jie Dou, Wei Mo, Shuai Liu, Bing Han, Yi Huang, Yanping Zhang, Mark A Varvares, Daniel F Hoft, Guangyong Peng

**Affiliations:** 1 Department of Internal Medicine, Division of Infectious Diseases, Allergy & Immunology, Saint Louis University School of MedicineSaint Louis, MO, USA; 2 Department of Laboratory Medicine, Women & Children's Health Care Hospital of LinyiLinyi, China; 3 Department of Surgery, Division of General Surgery, Saint Louis University School of MedicineSaint Louis, MO, USA; 4 Department of Otolaryngology-Head and Neck Surgery, Saint Louis University School of MedicineSaint Louis, MO, USA

**Keywords:** immunosenescence, regulatory T cells, toll-like receptor, tumor immunity, tumor microenvironment

## Abstract

Accumulating evidence suggests the immunosuppressive microenvironments created by malignant tumors represent a major obstacle for effective anti-tumor immunity. A better understanding of the suppressive mechanisms mediated by tumor microenvironments and the development of strategies to reverse the immune suppression are major challenges for the success of tumor immunotherapy. Here, we report that human tumor cells can induce senescence in naïve/effector T cells, exhibiting potent suppressive function *in vitro* and *in vivo*. We further show that tumor-derived endogenous cyclic adenosine monophosphate (cAMP) is responsible for the induction of T-cell senescence. Importantly, activation of TLR8 signaling in tumor cells can block the induction and reverse the suppression of senescent naïve and tumor-specific T cells *in vitro* and *in vivo*, resulting in enhanced anti-tumor immunity. These studies identify a novel mechanism of human tumor-mediated immune suppression and provide a new strategy to reverse tumor immunosuppressive effects for tumor immunotherapy.

## Introduction

Increasing evidence suggests that both CD4^+^ and CD8^+^ T cells play a critical role in cancer immunosurveillance and anti-tumor immunity, and manipulation of these immune cells to recognize and eradicate tumor cells is a promising strategy for treating patients with invasive and metastatic cancers. Numerous immunotherapeutic approaches have shown impressive results in preclinical animal and clinical studies, but the overall clinical responses of these immunotherapies have so far been insufficient to reproducibly eliminate tumors (Gajewski, 2006; Rosenberg *et al*, 2004). It has become clear that the tumor suppressive microenvironments created by malignant tumors are a major obstacle for effective anti-tumor immunity and successful tumor immunotherapy (Croci *et al*, 2007; Whiteside, 2008). Tumor cells can utilize different strategies to expand and recruit various types of suppressive tumor-infiltrating lymphocytes (TILs), including naturally occurring and adaptively induced regulatory T (Treg) cells, tolerogenic dendritic cells (DCs), tumor-derived macrophages and myeloid suppressor cells (MSCs) (Kiniwa *et al*, 2007; Kusmartsev & Gabrilovich, 2006; Peng *et al*, 2007; Roncarolo *et al*, 2006; Wei *et al*, 2005). In addition, tumor cells can secrete suppressive factors (IL-10, TGF-β and IDO), express immune inhibitory molecules (FasL and PD-L1), directly inhibit tumor-specific T-cell expansion and proliferation, or induce T-cell apoptosis (Croci *et al*, 2007; Dong *et al*, 2002; Uyttenhove *et al*, 2003; Whiteside, 2008). A better understanding of the molecular mechanisms involved in creating and sustaining the tumor-induced immune suppressive microenvironment is critical for the development of novel tumor vaccines and therapeutic strategies active against human cancers.

Cellular senescence was initially described in human fibroblasts with limited passage capacity in cell culture (Hayflick, 1965). Recent studies suggest that senescence also occurs in human T cells, causing age-associated dysregulation of the immune system during the normal aging process (Effros *et al*, 2005; Weng *et al*, 2009). Furthermore, accumulation of senescent CD8^+^ T cells has also been found in younger patients with chronic viral infections and patients with certain types of cancers, such as lung, breast, or head and neck cancers (Appay *et al*, 2000; Chen *et al*, 2009; Meloni *et al*, 2006; Montes *et al*, 2008; Tsukishiro *et al*, 2003; Wolfram *et al*, 2000). In addition, we more recently have demonstrated that human Treg cells can induce T-cell senescence (Ye *et al*, 2012, 2013). Senescent T cells develop significant phenotypic alterations, such as permanent loss of CD28 expression, cell cycle arrest, and up-regulation of the cell cycle-related genes p53, p21, and p16 (Effros *et al*, 2005; Ye *et al*, 2012, 2013). More importantly, senescent T cells have exhibited functional changes, including defective killing abilities and the development of potent negative regulatory functions (Appay *et al*, 2000; Chen *et al*, 2009; Cortesini *et al*, 2001; Montes *et al*, 2008; Vallejo, 2005; Ye *et al*, 2012). Thus, improved understanding of the molecular mechanisms responsible for the generation of senescent T cells in the tumor microenvironment and exploration of strategies capable of restoring the effector functions of senescent T cells are also critical for anti-tumor immunity.

Toll-like receptors (TLR) have recently been recognized as critical components of the innate immune system, acting as a link between innate and adaptive immunity. TLRs are also very important for regulating Treg cell function (Wang *et al*, 2008). We have demonstrated that human TLR8 signaling directly reversed the suppressive functions of naturally occurring CD4^+^ CD25^+^ Treg cells as well as tumor-derived CD4^+^, CD8^+^, and γδ Treg cells (Kiniwa *et al*, 2007; Peng *et al*, 2005, 2007). However, TLR signaling is not restricted to immune cells and is also detected on other types of cells, including tumor cells (Huang *et al*, 2008; Su *et al*, 2010). Several TLR ligands are now being developed as immunotherapeutic drugs or vaccine adjuvants for cancer treatment (Paulos *et al*, 2007; Smits *et al*, 2008). It has been shown that the TLR7 agonist imiquimod can directly induce apoptosis in basal cell carcinoma and patients with melanoma (Schon *et al*, 2003). Furthermore, TLR3 ligand Poly (I:C) and TLR9 ligand CpG can also mediate potent anti-tumor activity (Liang *et al*, 2010; Salaun *et al*, 2007). However, LPS and Loxoribine can favor tumor development and survival (Cherfils-Vicini *et al*, 2010; Huang *et al*, 2005). Understanding the unique signaling pathways in cancer mediated by different TLRs will be important for the development of TLR-based therapy for human cancer.

In this study, we identified a novel mechanism utilized by human tumor cells to induce immune suppression. Tumor cells convert naïve/effector T cells into senescent T cells, which mediate potent immunosuppressive activity. We demonstrated that tumor-derived endogenous cyclic adenosine monophosphate (cAMP) was responsible for tumor-induced senescence in T cells. In our efforts to explore the strategies for reversing tumor suppressive microenvironments, we further showed that TLR8 signaling can reverse tumor-induced T-cell senescence by blocking cAMP production in tumor cells. Finally, we explored the applicability of this strategy to achieve tumor immunotherapy *in vivo* in mouse models. We found that activation of TLR8 signaling in tumor cells can block tumor-induced conversion of naïve and tumor-specific T cells into senescent cells and reverse senescent T-cell-mediated suppression, resulting in enhanced anti-tumor immunity *in vivo*. These studies identify a novel mechanism related to human tumor-mediated immune suppression and provide a new strategy to reverse tumor immunosuppressive microenvironments for successful tumor immunotherapy.

## Results

### Tumor cells induce T-cell senescence with potent suppressive function

**Figure 1:**
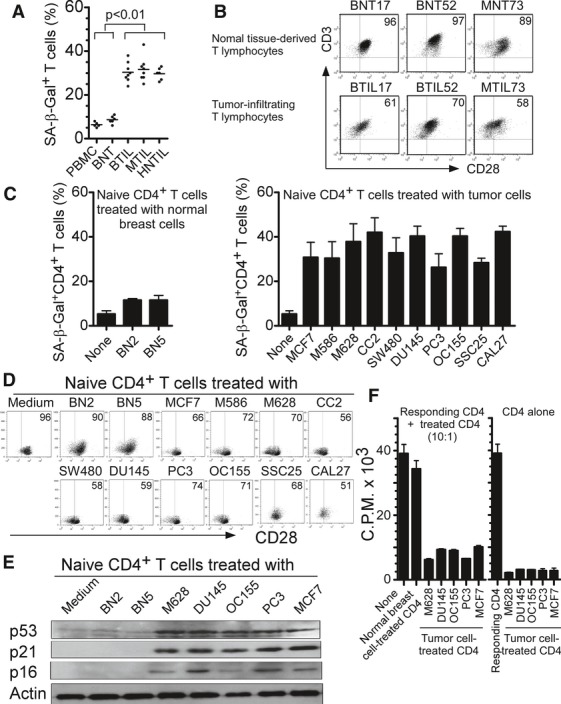
Tumor cells induce T-cell senescence A, B Increased SA-β-Gal-positive T-cell populations (A) and decreased CD28 expression (B) existed in TILs, compared with those in normal tissue-derived lymphocytes. CD3^+^ T cells were purified from the cultured tissue-infiltrating T cells from freshly digested tumor or normal tissues by microbeads and SA-β-Gal staining or CD28 expression was determined. BNT: normal breast tissue-derived T cells; BTIL, MTIL, and HNTIL: TIL obtained from breast cancer, melanoma, and head and neck cancers, respectively. C Tumor cell treatment significantly increased SA-β-Gal-positive T-cell populations in co-cultured naïve CD4^+^ T cells. However, naïve CD4^+^ T cells cultured in medium only or co-cultured with normal breast cells had little SA-β-Gal expression. Anti-CD3-activated naïve CD4^+^ T cells were co-cultured with different types of tumor cells or normal breast cells at ratio of 1:1 for 1 day. The treated CD4^+^ T cells were then separated and stained with SA-β-Gal staining reagents after culture for additional 3 days. Cell lines included: breast cancer (MCF7), melanoma (M586 and M628), colon cancer (SW480 and CC2), ovarian cancer (OC155), prostate cancer (DU145 and PC3), squamous cancers (SSC25 and CAL27), and normal breast cells (BN2 and BN5). D Decreased CD28 expression in naïve CD4^+^ T cells after culture with different types of tumor cell lines but not normal breast cells. Cell treatment and procedure were the same as in (C). CD28 expression in treated naïve CD4^+^ T cells were analyzed by flow cytometry. E Increased expression of cell cycle regulatory molecules p53, p21, and p16 in naïve T cells treated with tumor cells. Cell treatment and procedure were the same as in (C). Co-cultured naïve CD4^+^ T cells were purified and cell lysates were prepared for Western blot analyses. F Suppressive function of tumor-induced senescent T cells. Senescent CD4^+^ T cells induced by different types of tumor cell lines strongly inhibited the proliferation of responding CD4^+^ T cells. In contrast, naïve T cells treated or untreated with normal breast cells did not affect the proliferation of responding CD4^+^ T cells (left panel). Furthermore, senescent CD4^+^ T cells induced by different types of tumor cell lines did not proliferate themselves (right panel). Cell treatment and procedure were the same as in (C). Treated CD4^+^ T cells were purified, and the suppressive activity on responding CD4^+^ T-cell proliferation was evaluated using [^3^H]-thymidine incorporation assays. Data information: Data are representative of average of three independent experiments ± SD.

Accumulation of senescent CD8^+^CD28^null^ T cells has been found in patients with certain types of cancers, but the mechanisms responsible for the induction of these senescent T cells are still unclear (Meloni *et al*, 2006; Tsukishiro *et al*, 2003). To further determine the generality of development of senescent T cells as a potential form of tumor-mediated immune evasion (Montes *et al*, 2008), we first investigated the existence of senescent T-cell populations in tumor suppressive microenvironments. We generated TIL lines from fresh tumor tissues obtained from breast cancer, head and neck cancer, and patients with melanoma. In parallel, tumor-matched breast tissue-infiltrating T-cell lines from normal breast tissues were also generated. The percentages of SA-β-Gal, a specific biomarker to identify senescent human cells, positive cell populations in the TILs and normal breast tissue-infiltrating T lymphocytes were determined (Ye *et al*, 2012, 2013). We observed markedly elevated SA-β-Gal-positive T cells existing in TILs derived from melanoma, head and neck, and patients with breast cancer (over 25%) (Fig 1A). In contrast, SA-β-Gal-positive cell populations among the normal breast tissue-infiltrating T cells constituted less than 10%. In addition, this result was confirmed in directly purified TILs from the freshly digested tumor tissues (Supplementary Fig S1A). Since permanent loss of CD28 expression is the most consistent biological indicator of immunosenescent T cells (Effros *et al*, 2005; Vallejo, 2005; Ye *et al*, 2012), we determined whether TILs also down-regulated expression of the co-stimulatory molecule CD28. We found that both normal breast and melanoid tissue-infiltrating T lymphocytes expressed high levels of CD28. However, TILs derived from the matched patients with cancer significantly down-regulated CD28 expression, further confirming the existence of elevated senescent T-cell populations in the tumor microenvironments (Fig 1B). We then investigated whether tumor cells can directly induce T-cell senescence. Naïve CD4^+^ T cells purified from healthy donors and preactivated with anti-CD3 antibody were co-cultured with different types of tumor cell lines, including breast cancer (MCF7), melanoma (M586 and M628), colon cancer (SW480 and CC2), ovarian cancer (OC155), prostate cancer (DU145 and PC3), and squamous cancers (SSC25 and CAL27), or with normal breast cells (BN2 and BN5). After 1 day co-culture, the co-cultured CD4^+^ T cells were separated and continued in culture for 3 more days before analyses of senescence-associated phenotypes and function. Naïve CD4^+^ T cells cultured in medium only or co-cultured with normal breast cells only expressed minor levels of SA-β-Gal. In contrast, significantly increased SA-β-Gal-positive T-cell populations were induced in preactivated naïve CD4^+^ T cells after co-culture with different types of tumor cell lines, indicating that tumor cells can directly induce CD4^+^ T-cell senescence (Fig 1C and Supplementary Fig S1B). We also confirmed these results by co-culture of T cells with the primary tumor cells freshly purified from melanoma, breast and colon cancer tissues (Supplementary Fig S1C). In addition, a significant down-regulation of CD28 was induced in naïve CD4^+^ T cells after co-culture with the different types of tumor cell lines but not with normal breast cells (Fig 1D).

Senescence growth arrest is established and maintained by p53/p21 and/or p16/pRB tumor suppressor pathways (Campisi & d'Adda di Fagagna, 2007). We thus asked whether cell cycle regulatory molecules, including p53, p21, and p16, were involved in tumor-induced T-cell senescence. As expected, p16, p21, and p53 were significantly increased in naïve CD4^+^ T cells after co-culture with different types of tumor cell lines, while normal breast cell treatment did not induce this change in gene expression (Fig 1E). In addition to the measurement of morphologic and molecular changes, we examined tumor-induced senescent T cells for a permanent functional alteration. The suppressive activities of the senescent T cells induced by different types of tumor cells on the proliferation of responding CD4^+^ T cells were evaluated. We found that tumor-induced senescent CD4^+^ T cells strongly inhibited the proliferation of responding CD4^+^ T cells (Fig 1F), which is consistent with the findings from our and other groups that senescent CD8^+^ T cells have negative regulatory functions on immune responses induced by vaccination and transplantation (Chen *et al*, 2009; Cortesini *et al*, 2001; Ye *et al*, 2012). We obtained similar results that human tumor cell treatment can significantly induce senescence in naïve CD8^+^ T cells.

**Figure 2:**
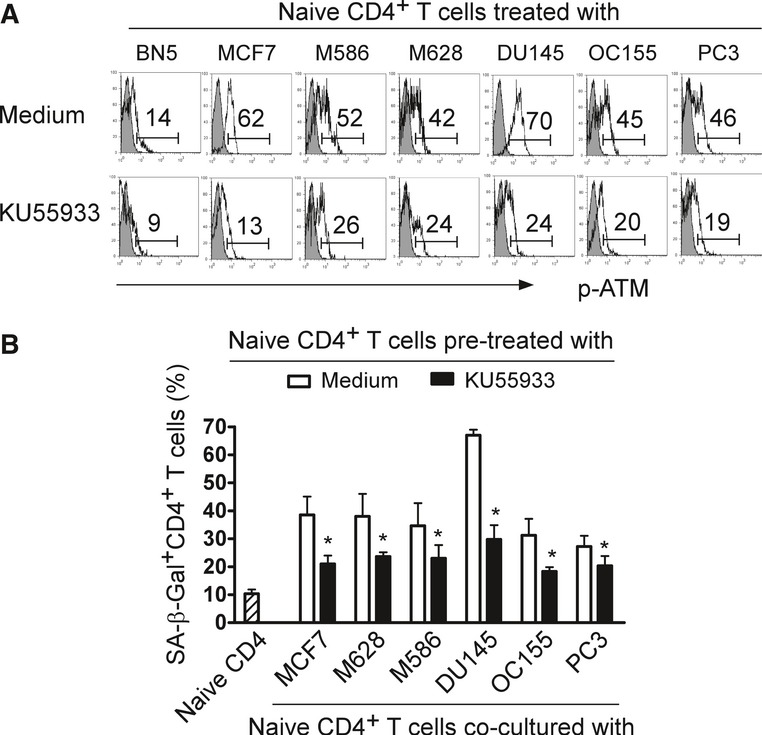
Tumor cell-induced T-cell senescence involves the DNA-damage response in senescent T cells Tumor cells induced phosphorylated activation of ATM in naïve CD4^+^ T cells. Furthermore, treatment with an ATM specific inhibitor KU55933 markedly suppressed the ATM phosphorylation in tumor-treated T cells. Anti-CD3 activated CD4^+^ T cells were pretreated with or without KU55933 (20 μM) for 1 day and then co-cultured with different types of tumor cells for another 1 day. The co-cultured CD4^+^ T cells were separated and the p-ATM expression determined after culture for 3 additional days using FACS analyses.Pretreatment of T cells with KU55933 significantly prevented the induction of T-cell senescence induced by different types of tumor cells. Cell treatment procedure was the same as in (A). SA-β-Gal expression in CD4^+^ T cells was determined with SA-β-Gal staining. Data shown are mean ± SD from three independent experiments, and paired *t*-test was performed. *P < 0.05, compared with the medium only group.

The induction of DNA damage is the key molecular process in senescent cells, which could be induced by telomere erosion and/or other forms of stress (Rodier *et al*, 2009; Van Nguyen *et al*, 2007). The nuclear kinase ataxia-telangiectasia mutated protein (ATM) is the chief inducer of the DNA-damage response. We found that significantly increased phosphorylated activation of ATM was induced in naïve CD4^+^ T cells treated with different types of tumors (Fig 2A). In addition, treatment with an ATM specific inhibitor (KU55933) dramatically suppressed the phosphorylation of ATM in tumor-treated T cells and prevented the induction of T-cell senescence induced by tumor cells (Fig 2B), further confirming the involvement of the DNA-damage response in tumor-induced T-cell senescence. Collectively, these results clearly indicate that human tumor cells can convert naïve T cells into senescent cells that possess potent suppressive activity and maintain a suppressive tumor microenvironment.

### Tumor-induced T-cell senescence is dependent on cell–cell contact, but independent of the suppressive cytokines IL-10 and TGF-β, and the inhibitory molecules PD-1 and IDO

**Figure 3:**
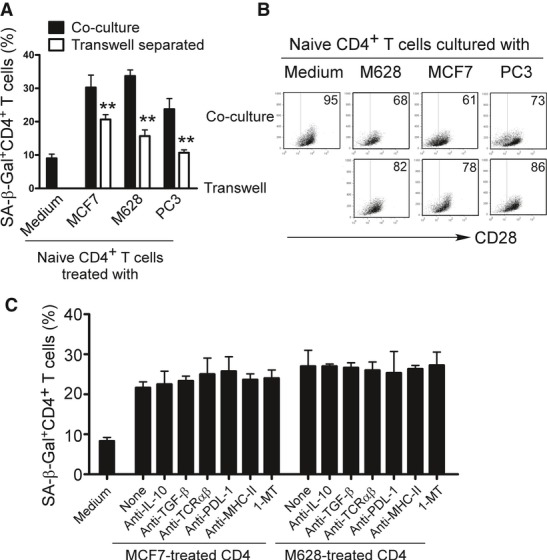
Tumor-induced T-cell senescence is dependent on cell–cell contact, but independent of known suppressive cytokines and inhibitory molecules Significant decrease of the SA-β-Gal-positive T-cell populations in naïve CD4^+^ T cells induced by tumor cells after separation by a Transwell system. Anti-CD3-activated naïve CD4^+^ T cells were either co-cultured with tumor cells as described in Fig 1 or were separated with tumor cells by a Transwell system in 24-well plates with pore size 0.4 μm insert chambers. SA-β-Gal expression was measured in the treated CD4^+^ T cells. Data shown are mean ± SD from three independent experiments, and paired *t*-test was used. **P < 0.01, compared with the naïve T cells in the co-culture system.Separation of naïve CD4^+^ T cells and tumor cells by a Transwell system alleviated the loss of CD28 expression in tumor-treated T cells. Cell treatment and procedure were the same as in (A). CD28 expression in treated naïve CD4^+^ T cells were analyzed by flow cytometry.Induction of T-cell senescence mediated by tumor cells cannot be prevented through the blockage of IL-10, TGF-β, TCRαβ, PDL-1 and MHC-class II and IDO pathways. Tumor cells and naïve CD4^+^ T cells were pretreated with various indicated neutralizing antibodies and the IDO inhibitor 1-MT and then co-cultured in the presence or absence of these neutralizing antibodies and 1-MT. The co-cultured CD4^+^ T cells were then separated and analyzed for SA-β-Gal expression.

In order to further identify molecules responsible for the induction of T-cell senescence mediated by tumor cells, we performed Transwell experiments to determine whether the induction of T-cell senescence by tumor cells was due to secreted molecules or required cell–cell contact. As shown in Fig 3A, the numbers of senescent T cells generated from naïve CD4^+^ T cells dramatically decreased when naïve CD4^+^ T cells were separated from tumor cells (MCF7, M628 and PC3) in a Transwell system, although the percentages of senescent T cells were still higher than those in the culture medium only group. Furthermore, the loss of CD28 expression in naïve CD4^+^ T cells induced by tumor cells was significantly alleviated when they were separated in the Transwell system (Fig 3B). To further confirm these results, we collected the culture supernatants from the different types of tumor cell lines and then determined the capacity of tumor cell supernatants to induce T-cell senescence. As expected, culture supernatants from three types of tumor cell lines induced minor or no senescence in cultured naïve T cells (Supplementary Fig S2A). These results suggest that cell–cell contact is required for the induction of T-cell senescence mediated by tumor cells.

Recent studies have shown that several potential molecules and pathways, such as suppressive cytokines IL-10 and TGF-β, as well as the inhibitor molecules PD-1 and IDO, are utilized by tumor cells to induce immunosuppression and immune escape (Croci *et al*, 2007; Uyttenhove *et al*, 2003; Whiteside, 2008). We investigated whether these molecules were also involved in the process of tumor-induced T-cell senescence. The capacity of tumor cells (MCF7 and M628) to induce senescence in naïve CD4^+^ T cells was investigated in the presence or absence of a panel of neutralizing antibodies against IL-10, TGF-β, TCRαβ, PDL-1, and MHC-class II, as well as the IDO inhibitor, 1-methyl-D-tryptophan (1-MT). However, none of the neutralizing antibodies or 1-MT blocked the induction of T-cell senescence and loss of CD28 induced by tumor cells (Fig 3C and Supplementary Fig S2B). These data clearly suggest that the mechanism responsible for tumor-induced T-cell senescence is independent of IL-10, TGF-β, TCRαβ, MHC, PDL-1, and IDO molecules or pathways.

### Tumor-derived cAMP is responsible for the tumor cell-induced T-cell senescence

**Figure 4:**
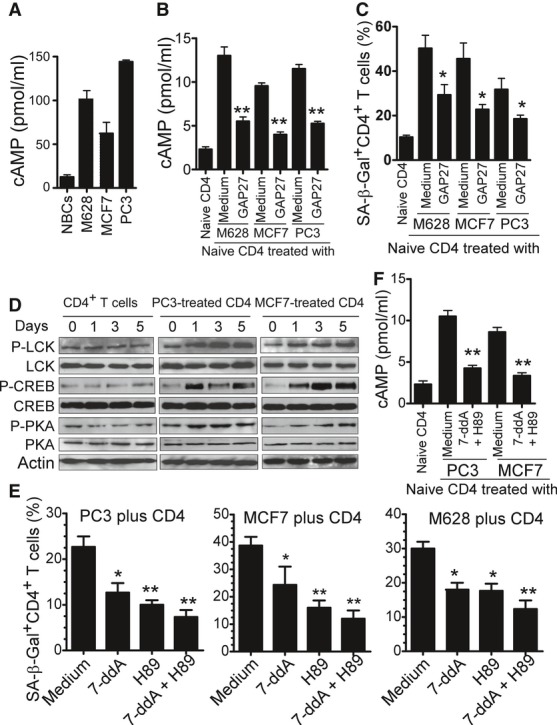
Tumor-derived endogenous cAMP is a key mediator inducing T-cell senescence cAMP levels in M628, PC3, and MCF7 tumor cells were significantly higher than those in normal breast cells. Concentrations of cAMP in cell lysates were determined by ELISA.cAMP levels in CD4^+^ T cells were significantly increased after co-culture with M628, MCF7, or PC-3 tumor cells, whereas its levels in CD4^+^ T cells were dramatically decreased if the tumors and T cells were co-cultured in the presence of the gap junction inhibitory peptide GAP27. Tumor cells were co-cultured with anti-CD3-activated CD4^+^ T cells in the presence or absence of GAP27 (300 μM) for 1 day. The treated T cells were separated and the cAMP levels determined after culture for additional 3 days.Blocking gap junction formation with GAP27 significantly prevented tumor-induced CD4^+^ T-cell senescence. Cell treatment and procedure were the same as in (B). SA-β-Gal expression was measured in the treated CD4^+^ T cells.PC3 and MCF7 tumor cells induced phosphorylation of LCK, CREB, and PKA in senescent CD4^+^ T cells. Anti-CD3-activated CD4^+^ T cells were co-cultured with MCF7 or PC3 for 0, 1, 3, and 5 days. Co-cultured naïve CD4^+^ T cells were purified and then lysates prepared for Western blot analyses.Inhibition of cAMP signaling by specific pharmacological inhibitors significantly reversed T-cell senescence induced by tumor cells. Anti-CD3 preactivated naïve CD4^+^ T cells were co-cultured with tumor cells in the presence or absence of inhibitors 7-ddA (320 μM), or H89 (20 μM) or the combination for 1 day. Co-cultured CD4^+^ T cells were separated, and SA-β-Gal expression was determined after culture for an additional 3 days.Pretreatment of tumor cells with the 7-ddA and H89 markedly decreased cAMP levels in co-cultured CD4^+^ T cells. MCF7 or PC-3 cells were pretreated with or without 7-ddA (320 μM) and H89 (20 μM) for 1 day, washed, and then co-cultured with anti-CD3 activated CD4^+^ T cells for 1 day. The co-cultured CD4^+^ T cells were purified and cAMP levels determined. Data information: Data shown are mean ± SD from three independent experiments and paired *t*-test was performed between groups. *P < 0.05 and **P < 0.01, compared with the medium only group (B, C, and F), or with the group not treated with inhibitors (E).

Another important strategy utilized by tumor cells is to create a hypoxic microenvironment, resulting in accumulation of adenosine and cAMP within the tumor sites (Ohta *et al*, 2006; Sitkovsky *et al*, 2008). These hypoxia-derived metabolites are potent immunosuppressors that can protect tumor cells from anti-tumor immune responses mediated by tumor-specific CD4^+^ and CD8^+^ T cells (Ohta *et al*, 2006; Sitkovsky *et al*, 2008). Furthermore, cAMP is also a key component involved in Treg-mediated suppression, and a potent inhibitor of IL-2 production and subsequent CD4^+^ T-cell proliferation (Bopp *et al*, 2007; Vang *et al*, 2001). These studies prompted us to test whether tumor-induced hypoxia-derived cAMP was responsible for the induction of T-cell senescence. Notably, high concentrations of cAMP were detected in primary and tumor cell lines of melanoma, prostate and breast cancer cells, whereas the concentration of this seconder messenger in normal breast cells was very low (Fig 4A). Furthermore, after 1 day co-culture with tumor cells, the co-cultured naïve CD4^+^ T cells also displayed a significant increase in cAMP level (Fig 4B). These data suggest that high concentrations of endogenous cAMP exist in tumor cells and in tumor-induced senescent T cells. Treg cells have been shown to be able to transfer cAMP through gap junctions to their responder T cells (Bopp *et al*, 2007). Given that maximal tumor-induced T-cell senescence is dependent on cell–cell contact, we reasoned that tumor cells may utilize a mechanism similar to Treg cells to transfer cAMP to their targeted T cells, resulting in the induction of T-cell senescence. To test this possibility, we first evaluated the capacity of tumor cells to transfer a permeant dye calcein AM to T cells; this dye has been reported to only be transferred from donor to recipient cells via gap junctions (Bopp *et al*, 2007; Moreno-Fernandez *et al*, 2011). Anti-CD3 activated CD4^+^ T cells were co-cultured with calcein AM-labeled MCF7, M628 and PC3 tumor cells for 1 to 3 days, and calcein AM positive T cells were determined. As shown in Supplementary Fig S3A, significantly increased percentages of calcein AM positive T cells were detected in the co-cultured T cells, suggesting a transfer of calcein AM from tumor cells to these responder T cells. Furthermore, GAP27, a peptide known to specifically block gap junction formation, dramatically decreased the calcein AM positive T-cell populations in CD4^+^ T cells co-cultured with calcein AM-labeled tumor cells (Supplementary Fig S3B). We then determined whether tumor cells can transfer cAMP to T cells through gap junctions. As expected, blockage of gap junction formation with GAP27 also significantly decreased the cAMP levels in naïve CD4^+^ T cells co-cultured with tumor cells (Fig 4B). Importantly, blockage of gap junction formation with GAP27 also markedly prevented the induction of T-cell senescence mediated by tumor cells (Fig 4C). It has been reported that cAMP inhibits T-cell activation through a protein kinase A (PKA) type I–COOH-terminal Src kinase (Csk)–LCK inhibitory pathway (Tasken & Stokka, 2006; Vang *et al*, 2001). We next investigated the cAMP signaling involved in tumor-induced T-cell senescence. Anti-CD3-preactivated CD4^+^ T cells were co-cultured with or without tumor cells for different time periods, and the expression and phosphorylation levels of protein tyrosine kinase LCK and key cAMP downstream regulatory molecules, including PKA and the cAMP-responsive element binding protein (CREB), were measured. We found that PC3 and MCF7 tumor cells dramatically increased phosphorylation of LCK, PKA, and CREB in senescent CD4^+^ T cells, suggesting that tumor cell treatment induced LCK inhibitory signaling in senescent T cells (Fig 4D) (Tasken & Stokka, 2006; Vang *et al*, 2001).

To further investigate the functional role of cAMP in tumor-induced T-cell senescence, anti-CD3 preactivated CD4^+^ T cells were co-cultured with tumor cells in the presence or absence of specific cAMP pharmacological inhibitors [7-ddA (320 μM), H89 (20 μM) or the combination]. The co-cultured CD4^+^ T cells were separated and SA-β-Gal expression was investigated. As shown in Fig 4E and Supplementary Fig S4A, inhibition of the cAMP signaling by specific pharmacological inhibitors significantly reversed tumor-induced senescence in naïve T cells. To exclude the possibility that the cAMP inhibitors might directly target the co-cultured T cells rather than tumor cells, tumor cell lines were pretreated with the cAMP inhibitors. After extensive washes, these pretreated tumor cells were co-cultured with anti-CD3 preactivated CD4^+^ T cells, and their capacity to induce T-cell senescence determined. We obtained very similar results as shown in the co-culture system, suggesting that these inhibitors prevent T-cell senescence through direct effects on tumor cells (Supplementary Fig S4B). Notably, cAMP levels in CD4^+^ T cells markedly decreased if the tumor cells were pretreated with the pharmacological inhibitors of 7-ddA and H89 (Fig 4F). In addition, pretreatment of tumor cells with forskolin, a cAMP-elevating agent (Bopp *et al*, 2007), or 3-isobutyl-1-methylxanthine (IBMX), an inhibitor for cAMP monophosphate phosphodiesterase (PDE) (Grader-Beck *et al*, 2003), significantly promoted the induction of naïve T-cell senescence co-cultured with the pretreated tumor cells (Supplementary Fig S5A). Forskolin treatment of naïve T cells can directly induce T-cell senescence (Supplementary Fig S5B).

To identify the molecular links between cAMP and tumor-induced T-cell DNA-damage response and senescence, phosphorylated activation of ATM in T cells co-cultured with different tumor cells were determined in the presence or absence of specific pharmacological inhibitors for cAMP signaling. We observed that inhibition of the cAMP in tumor cells by 7-ddA, H89, or the combination, significantly suppressed the phosphorylation of ATM in tumor-treated T cells (Supplementary Fig S6A). Furthermore, Forskolin treatment of naïve T cells can also markedly increase the ATM phosphorylation (Supplementary Fig S6B). To further confirm that cAMP-induced PKA-CREB pathway involves T-cell senescence, we found that treatment with the inhibitors 7-ddA and H89 dramatically decreased phosphorylation of CREB in CD4^+^ T cells co-cultured with tumor cells (Supplementary Fig S7A). In addition, knockdown of CREB expression in CD4^+^ T cells with specific siRNA significantly prevented tumor cell-induced CD4^+^ T-cell senescence (Supplementary Fig S7B). Taken together, our results clearly indicate that tumor-derived endogenous cAMP is an important mediator for senescence induction in responder T cells.

### Direct effects of TLR ligands on tumor-induced T-cell senescence

**Figure 5:**
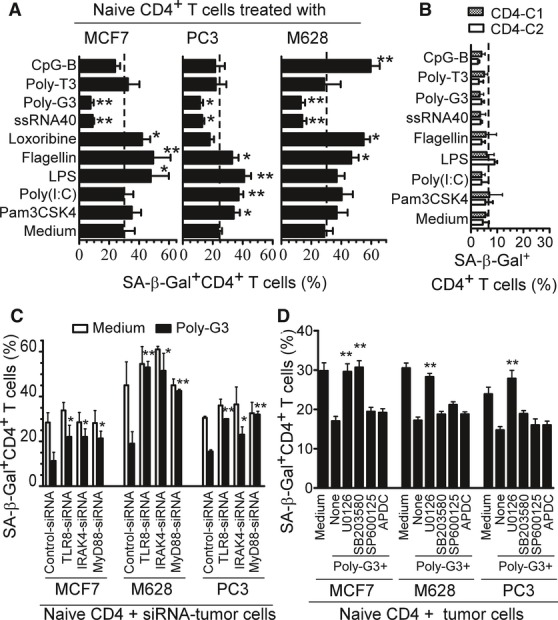
TLR8 signaling prevents the induction of CD4^+^ T-cell senescence mediated by tumor cells TLR8 ligands Poly-G3 and ssRNA40 markedly reversed the ability of tumor cells to induce naïve CD4^+^ T-cell senescence. MCF7, PC3, and M628 cells were co-cultured with anti-CD3 activated CD4^+^ T cells in the presence or absence of the indicated TLR ligands for 1 day. The treated CD4^+^ T cells were then separated and SA-β-Gal expression determined after culture for additional 3 days. Poly-T3 (3 μg/ml) served as a control.TLR ligands did not induce senescence in CD4^+^ T cells. Preactivated CD4^+^ T cells freshly purified from two healthy donors were cultured in the presence of the indicated TLR ligands for 3 days and SA-β-Gal-positive CD4^+^ T cells determined.Knockdown of TLR8, MyD88, or IRAK4 in MCF7, M628, or PC3 tumor cells blocked the reversal of tumor-induced CD4^+^ T-cell senescence mediated by Poly-G3. Tumor cells were transfected with lenti-shRNAs specific for TLR8, MyD88, or IRAK4 molecules. Transduced (GFP^+^) tumor cells were purified by FACS sorting and co-cultured with anti-CD3 activated CD4^+^ T cells in the presence or absence of Poly-G3 for 1 day. The treated CD4^+^ T cells were then separated, and the SA-β-Gal expression was determined after culture for additional 3 days.Pretreatment of PC3 or M628 tumor cells with the inhibitor U0126, and MCF7 cells with both inhibitors U0126 and SB203580, significantly blocked the Poly-G3-mediated reversal of tumor-induced T-cell senescence. Tumor cells were pretreated for 3 days with MAPK inhibitors U0126 (10 μM), SB203580 (10 μM) and SP600125 (10 μM), or the NF-κB inhibitor APDC (10 μM), and Poly-G3 was added into co-cultures on day 3. The pretreated tumor cells were co-cultured with anti-CD3-activated CD4^+^ T cells at ratio of 1:1 for 1 day. The treated T cells were separated and cultured for additional 3 days, and then SA-β-Gal expression assessed. Data information: Data are average (A) or mean (C, D) of three independent experiments ± SD, and paired *t*-test was used between groups. *P < 0.05 and **P < 0.01, compared with the group not treated with TLR ligand (A), with the group transfected with control shRNA (C), or with the group not treated with inhibitor (D).

Increasing evidence suggests that chronic infection and inflammation are significant environmental factors for tumorigenesis (Karin *et al*, 2006). Given the facts that studies from multiple preclinical animal models and clinical trials have shown that either promotion or inhibition of tumor survival and growth was mediated by different TLR stimulations in tumor cells (Huang *et al*, 2008; Paulos *et al*, 2007; Smits *et al*, 2008), we reasoned that TLR signaling may affect tumor-induced T-cell senescence and TIL function in the tumor microenvironments. To test our hypothesis, we first confirmed the TLR (TLR1-9) expression profiles in different types of tumor cells (primary or cell lines) (Supplementary Fig S8). We next sought to determine whether TLR signaling in tumor cells influenced the ability of tumor cells to induce T-cell senescence. MCF7, PC3, and M628 tumor cell lines were co-cultured with anti-CD3 activated CD4^+^ T cells in the presence or absence of different TLR ligands, and senescence induction in the treated CD4^+^ T cells was determined. As shown in Fig 5A, we observed that TLR8 ligands Poly-G3 and ssRNA40, but not ligands for other TLRs, markedly reversed the senescence induction in naïve CD4^+^ T cells mediated by the three tumor cell lines. However, other TLR ligands promoted the induction of T-cell senescence mediated by tumor cells, which varied among the tumor cell lines. T-cell senescence was promoted in MCF7 cells treated with LPS (TLR4), Flagellin (TLR5) and Loxoribine (TLR7); PC3 cells treated with Pam3CSK4 (TLR2), Poly (I:C)(TLR3), LPS (TLR4), and Flagellin (TLR5); and M628 cells treated with Flagellin (TLR5), Loxoribine (TLR7), and CPG-B (TLR9). To exclude the possibility that these TLR ligands could directly induce T-cell senescence, preactivated CD4^+^ T cells freshly purified from two healthy donors were cultured in the presence of different TLR ligands and then examined for senescence induction. Direct treatment of CD4^+^ T cells with these TLR ligands had no effect on the induction of T-cell senescence (Fig 5B). These results suggest that inflammation triggered by TLR signaling can directly influence tumor-induced senescence in tumor microenvironments, and the effects are variable depending on different TLR signaling and tumor types.

### TLR8 signaling reverses tumor-induced T-cell senescence via down-regulation of cAMP in tumor cells

We have identified a novel mechanism that links TLR8 signaling to the inhibition of Treg cell function (Peng *et al*, 2005). We found that synthetic Poly-G3 and natural TLR8 ligand (ssRNA40) completely reversed the suppressive functions of naturally occurring CD4^+^CD25^+^ Treg, and tumor-derived CD4^+^, CD8^+^, and γδ Treg cells (Kiniwa *et al*, 2007; Peng *et al*, 2005, 2007). Importantly, in the current studies, we also observed that Poly-G3 and natural ssRNA40 can block the induction of T senescence mediated by tumor cells (Fig 5A). These results prompted us to further confirm whether the reversal of tumor-induced T-cell senescence mediated by Poly-G3 and ssRNA40 occurs through TLR8 signaling, using a loss-of-function strategy with siRNA (Peng *et al*, 2005, 2007). MCF7, PC3, and M628 tumor cells were transfected with lenti-shRNAs specific for TLR8, MyD88, or IRAK4 molecules, or control lenti-shRNA, and then their ability to induce T-cell senescence in the presence of Poly-G3 was determined. As shown in Fig 5C, knockdown of TLR8, MyD88, and IRAK4 in tumor cells significantly blocked the Poly-G3-mediated reversal of tumor-induced CD4^+^ T-cell senescence. These results confirmed that TLR8 signaling can prevent the induction of T-cell senescence mediated by tumor cells. To further investigate the unique downstream signaling pathway of TLR8 responsible for the control of the capacity of tumor-induced T-cell senescence, we still utilized loss-of-function strategies involving both specific pharmacological inhibitors and siRNA to block NF-κB and MAPK signaling pathways (JNK, ERK1/2 and p38) in tumor cells. As shown in Fig 5D, pretreatment of all three tumor cells (MCF7, PC3, or M628) with the ERK1/2 pharmacological inhibitor (U0126) significantly blocked the Poly-G3-mediated reversal of tumor-induced T-cell senescence. Furthermore, pretreatment of MCF-7 cells with the p38 inhibitor SB203580 also prevented Poly-G3 from reversing the induction of T-cell senescence. However, pretreatment of these tumor cells with the inhibitors SP600125 (for JNK) and ADPC (for NF-κB) did not alter the Poly-G3-mediated reversal of T-cell senescence induced by tumor cells. These results were further confirmed by knockdown of the NF-κB and the MAPK signaling pathways in the tumor cells with lenti-shRNAs specific for ERK1/2, p38α, JNK1, or IKKα (Supplementary Fig S9). These results identify the key adaptor molecules involved in TLR8 signaling, including MyD88, IRAK4, ERK1/2, and p38, but not JNK and IKK, as necessary for reversal of tumor-induced responder T-cell senescence.

**Figure 6:**
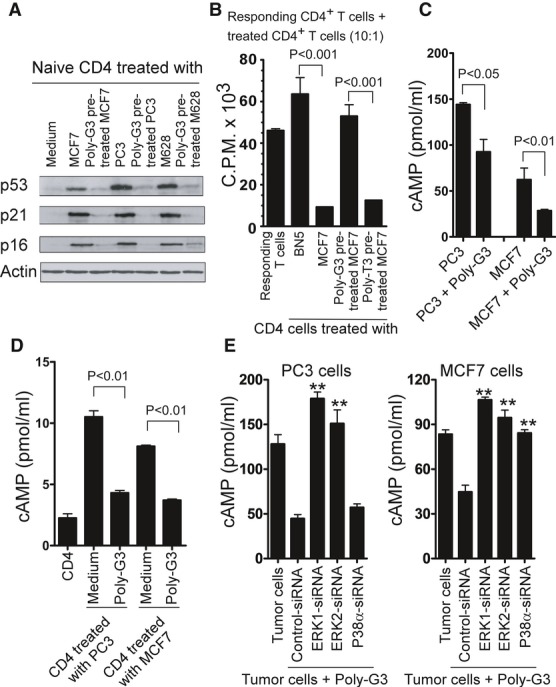
TLR8 signaling reverses tumor-induced T-cell senescence via down-regulation of cAMP in tumor cells A Pretreatment of tumor cells with Poly-G3 down-regulated the expression of p53, p21, and p16 in tumor-induced senescent CD4^+^ T cells. CD4^+^ T cells were co-cultured with Poly-G3-pretreated or untreated tumor cells (MCF7, PC3, or M628) for 1 day. The cultured CD4^+^ T cells were separated, and p53, p21, and p16 expression was determined using Western blot analysis after culture for 3 additional days. B Pretreatment of MCF7 cells with Poly-G3, but not with Poly-T3 (control), significantly reversed the suppressive activity on the responding T-cell proliferation mediated by tumor-treated naïve CD4^+^ T cells. Naive CD4^+^ T cells were co-cultured with BN5 or MCF7 cells pretreated with or without Poly-G3 for 1 day. Treated CD4^+^ were separated, and the suppressive activities on responding CD4^+^ T-cell proliferation were evaluated using [^3^H]-thymidine incorporation assays. C, D Poly-G3 treatment significantly decreased cAMP levels in tumor cells (C) and in tumor-induced senescent CD4^+^ T cells (D). MCF7 or PC-3 cells were co-cultured with Anti-CD3-activated CD4^+^ T cells for 1 day in the presence or absence of Poly-G3 (3 μg/ml). cAMP levels in tumor cells and in co-cultured CD4^+^ T cells were detected. E Knockdown of ERK1/2 and p38α in MCF7, and ERK1/2 in PC3 tumor cells significantly blocked the Poly-G3-mediated down-regulation of cAMP levels in tumor cells. Tumor cells were transfected with lenti-shRNAs specific for ERK1, ERK2 or p38α molecules or control shRNA. Transduced (GFP^+^) tumor cells were purified by FACS sorting and cultured for 1 day in the presence of Poly-G3 (3 μg/ml). cAMP levels in tumor cells were detected. Data information: Data shown are mean of three independent experiments ± SD, and paired *t*-test was performed between groups. **P < 0.01, compared with the group transfected with control shRNA (E).

In addition to evaluation of SA-β-Gal expression in tumor-treated CD4^+^ T cells, we investigated the effects of TLR8 signaling on the expression of other senescence-associated markers and function of tumor-induced senescent T cells. CD4^+^ T cells were co-cultured with tumor cells (MCF7, PC3 or M628) pretreated with or without Poly-G3, and the expression of cell cycle molecules and the suppressive effects on T-cell proliferation were determined using Western blot and [^3^H]-thymidine incorporation assays, respectively. Pretreatment of MCF7, PC3, and M628 tumor cells with Poly-G3 markedly down-regulated the expression of p53, p21, and p16 in tumor-induced senescent CD4^+^ T cells (Fig 6A). In addition, pretreatment of MCF7 cells with Poly-G3, but not control Poly-T3, significantly reversed the suppressive activity of tumor-induced senescent CD4^+^ T cells on the responding T-cell proliferation (Fig 6B).

Since our studies have demonstrated that tumor-derived endogenous cAMP is an important mediator for tumor-induced T-cell senescence, we next investigated whether the preventive effects of the TLR8 ligand Poly-G3 on tumor-induced T-cell senescence were due to the decreases in cAMP levels in tumor cells. Indeed, Poly-G3 treatment significantly decreased the cAMP levels in tumor cells (Fig 6C). Notably, pretreatment of tumor cells with Poly-G3 also markedly decreased the cAMP levels in T cells co-cultured with tumor cells (Fig 6D). We then determined whether blockage of TLR8 signaling, responsible for the reversal of tumor-induced T-cell senescence, can also affect endogenous cAMP production in tumor cells after treatment with Poly-G3. MCF7 and PC3 tumor cells were transfected with lenti-shRNAs specific for ERK1, ERK2, p38α, or control lenti-shRNA, and then cAMP levels produced by Poly-G3-treated tumor cells were determined. As shown in Fig 6E, knockdown of ERK1/2 and/or p38α in MCF7, and ERK1/2 in PC3 tumor cells, significantly blocked the Poly-G3-mediated down-regulation of cAMP levels in tumor cells. In contrast, control lenti-shRNA did not prevent Poly-G3-mediated decreases of cAMP levels in tumor cells. Collectively, our studies clearly demonstrate that TLR8 signaling can reverse tumor-induced T-cell senescence through the down-regulation of endogenous cAMP levels in tumor cells.

### Prevention of tumor-induced T-cell senescence in naïve T cells by TLR8 signaling *in vivo*

**Figure 7:**
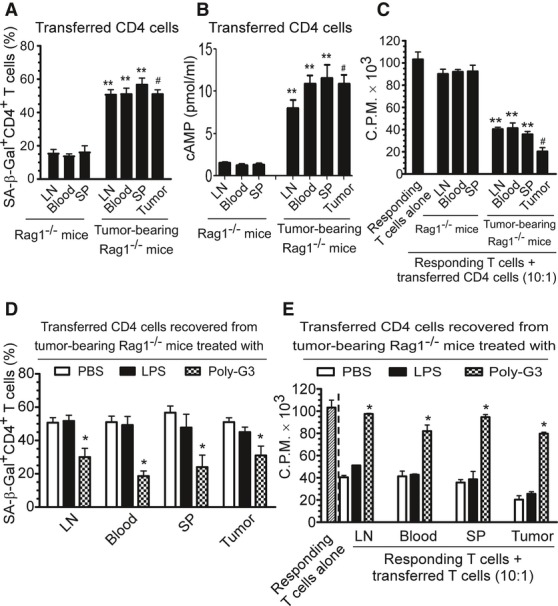
Human melanoma cells induce naïve CD4^+^ T-cell senescence *in vivo* A Increased SA-β-Gal-positive cell populations were markedly induced in preactivated human CD4^+^ T cells after adoptive transfer into the tumor 586mel-bearing *Rag1*^−/−^ mice but not in control *Rag1*^−/−^ mice. Human naïve CD4^+^ T cells (5 × 10^6^/mouse) were preactivated with anti-CD3 antibody and adoptively transferred into control *Rag1*^−/−^ mice or human 586mel-bearing *Rag1*^−/−^ mice. The transferred human CD4^+^ T cells were isolated and recovered from the blood, LNs and SPs, as well as tumor tissues (tumor-bearing mice) at 12 days post-injection for subsequent SA-β-Gal staining. B cAMP levels were significantly increased in CD4^+^ T cells after adoptive transfer into the tumor 586mel-bearing *Rag1*^−/−^ mice but not in control *Rag1*^−/−^ mice. Cell treatment and adoptive transfer procedure were identical as in (A). C The transferred human CD4^+^ T cells isolated and recovered from different organs and tumors in tumor 586mel-bearing mice had potent suppressive activity on the proliferation of responding CD4^+^ T cells using ^3^H-thymidine incorporation assays. D, E Intratumoral injection of Poly-G3 (50 μg in 100 μl PBS/mouse), but not LPS (10 μg in 100 μl PBS/mouse) or PBS (100 μl/mouse), can significantly block the induction of senescence and reverse the suppressive activity in transferred naïve CD4^+^ T cells in human 586mel-bearing mice. Cell preparation and injection procedures were the same as in (A). The transferred human CD4^+^ T cells in different organs were isolated at 12 days post-injection for subsequent SA-β-Gal staining (D) and ^3^H-thymidine incorporation assays (E). Data information: Results shown are mean ± SD (*n* = 5 mice per group). **P < 0.01, compared with the respective CD4^+^ T-cell group recovered from control *Rag1*^−/−^ mice (A–C); ^#^*P *< 0.01, compared with the average levels of CD4^+^ T-cell groups recovered from control *Rag1*^−/−^ mice (A–C); *P < 0.05 compared with the PBS group (D, E) using unpaired *t*-test.

We next explored whether human tumor cells can also convert naive T cells into senescent T cells with potent suppressive activity *in vivo,* using our previously established adoptive transfer model (Peng *et al*, 2005, 2007; Ye *et al*, 2012). Human 586mel tumor cells were subcutaneously injected into *Rag1*^−/−^ mice (lacking T and B cells) to establish a human tumor 586mel-bearing mouse model. Anti-CD3 preactivated naïve CD4^+^ T cells were adoptively transferred into control or 586mel tumor-bearing *Rag1*^−/−^ mice through intravenous injection. Blood, lymph nodes (LN), spleens (SP), and tumor tissues were harvested at 12 days post T-cell injection, and the transferred human CD4^+^ T cells were isolated to determine their levels of cAMP, senescence, and suppressive activity. As shown in Fig 7A, appropriately 10–15% of adoptively transferred preactivated CD4^+^ T cells became senescent T cells as identified by SA-β-Gal staining in control *Rag1*^−/−^ mice at 12 days post-injection. However, significant increases in senescent T-cell populations were induced in preactivated naïve CD4^+^ T cells in 586mel tumor-bearing mice (over 50%), indicating that human tumor cells can induce naive T-cell senescence *in vivo*. As expected, increased cAMP levels in the recovered CD4^+^ T cells were also observed in 586mel tumor-bearing mice (Fig 7B). Notably, cAMP levels and percentages of senescent T-cell populations isolated from different organs were very similar. In addition, intra-tumoral injection of 7-ddA and H89 can significantly decrease the cAMP levels in transferred CD4^+^ T cells in 586mel-bearing mice (Supplementary Fig S10). We next determined the suppressive activity of the recovered CD4^+^ T cells on the proliferation of responding T cells using ^3^H-thymidine incorporation assays. We found that the purified CD4^+^ T cells from different organs previously transferred into 586mel-bearing mice potently suppressed the proliferation of responding CD4^+^ T cells *in vitro*. In contrast, purified CD4^+^ T cells previously transferred into control *Rag1*^−/−^ mice did not have any suppressive activity (Fig 7C).

Our *in vitro* studies have shown that treatment of tumor cells with TLR8 ligands can reverse tumor cell-induced senescence. Thus, we investigated whether we can prevent the induction of T-cell senescence mediated by tumor cells *in vivo* by activation of TLR8 signaling in the adoptive transfer model. Preactivated naïve CD4^+^ T cells were adoptively transferred into 586mel-bearing *Rag1*^−/−^ mice through intravenous injection following the same procedures as above. Concomitantly, TLR8 ligand Poly-G3 was intratumorally injected on days 1, 4, 7, and 10 after adoptive transfer of CD4^+^ T cells. TLR4 ligand LPS and PBS treatment groups were included as controls. We then investigated the senescence induction and suppressive activity of the recovered CD4^+^ T cells in organs and tumors from different treatment groups at 12 days post T-cell transfer. We found high percentages of senescent cells existing in the transferred activated CD4^+^ T cells recovered from 586mel-bearing mice treated with PBS, consistent with the results observed in Fig 7A. In contrast, we observed that intratumoral injection of Poly-G3 significantly blocked the induction of senescence in transferred CD4^+^ T cells (Fig 7D). However, there was no observed effect on senescence induction in transferred naïve CD4^+^ T cells if the tumor was treated with LPS, although our *in vitro* data showed that LPS treatment on some tumor cells, such as MCF7 and PC3 cells, induced increased senescent cell populations in treated naïve CD4^+^ T cells (Fig 5A). Furthermore, treatment of tumor cells with Poly-G3, but not LPS or PBS, markedly reversed the suppressive activity of senescent CD4^+^ T cells induced by tumor cells in 586mel-bearing mice (Fig 7E). Notably, we also evaluated the effects of different concentrations (10, 20, and 50 μg/mice) of LPS treatment on tumor cells and did not observe any prevention of senescence induction or reversal of suppressive activity in transferred naïve T cells recovered from the tumor-bearing mice. These results collectively indicate that human tumor cells can convert responder naïve T cells into senescent T cells with suppressive functions both *in vitro* and *in vivo* and that TLR8 signaling activation in tumor cells can prevent tumor-mediated induction of T-cell senescence and subsequent immune suppression.

### Blockage of tumor-induced senescence in tumor-specific effector T cells enhances anti-tumor immunity *in vivo* in an adoptive transfer therapy model

**Figure 8:**
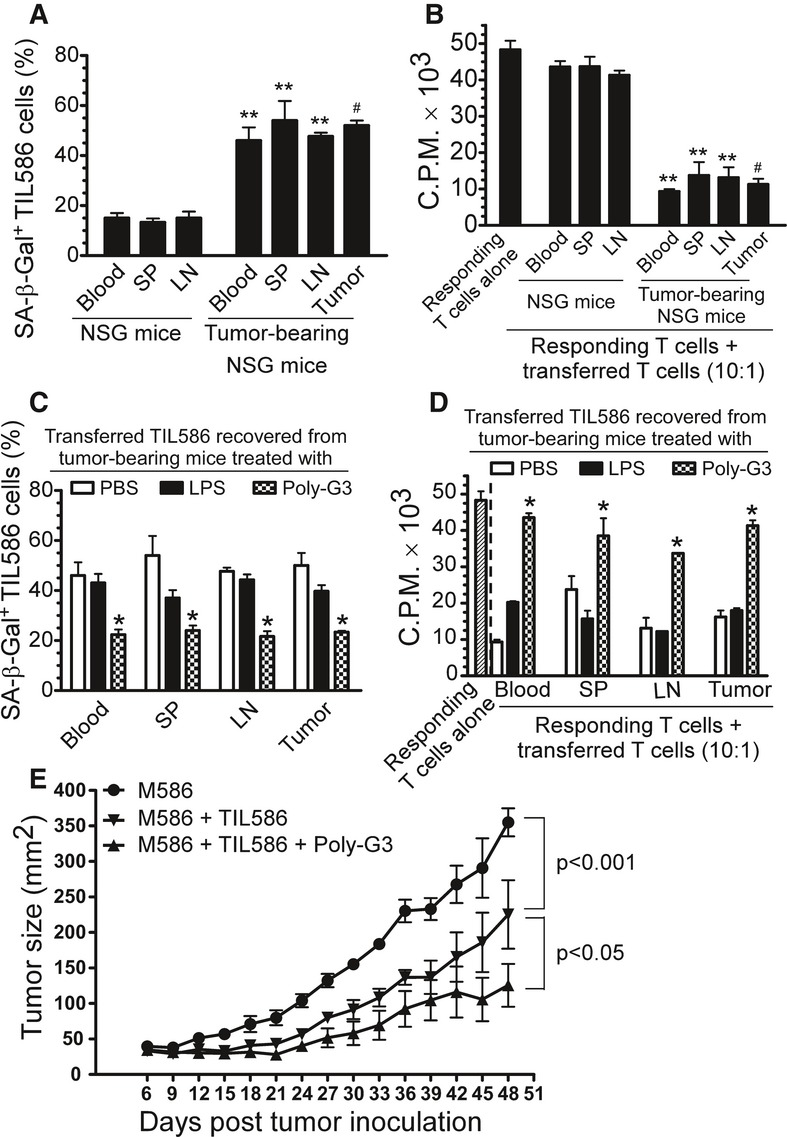
Enhancement of anti-tumor immunity mediated by tumor-specific CD8^+^ T cells protected against tumor-induced senescence via TLR8 signaling *in vivo* A SA-β-Gal-positive cell populations were markedly induced in human tumor-specific CD8^+^ TIL586 cells after adoptive transfer into the tumor 586mel–bearing NOD-scid *Il2rg*^null^ (NSG) mice but not into control NSG mice. Human CD8^+^ TIL586 cells (5 × 10^6^/mouse) were adoptively transferred into control or human 586mel-bearing NSG mice. Blood, LNs and SPs, as well as tumor tissues (tumor-bearing mice) were harvested at 12 days post-injection. The transferred human CD8^+^ TIL586 were isolated and recovered for subsequent SA-β-Gal staining. B The purified CD8^+^ TIL586 cells transferred into 586mel-bearing mice became suppressive T cells and had suppressive activity on the proliferation of responding CD4^+^ T cells. Cell treatment and adoptive transfer procedure were identical as in (A). The transferred human CD8^+^ TIL586 were isolated and recovered for subsequent ^3^H-thymidine incorporation assays. C, D Intratumoral injection of Poly-G3 but not LPS can significantly block the induction of senescence and reverse the suppressive activity in transferred CD8^+^ TIL586 cells in human 586mel-bearing mice. Cell preparation and injection procedures were the same as in (A). PBS (100 μl/mouse), LPS (10 μg in 100 μl PBS/mouse), or Poly-G3 (50 μg in 100 μl PBS/mouse) was injected into tumors on days 1, 4, 7, and 10 after adoptive transfer of CD8^+^ TIL586 cells into the mice. The transferred human CD8^+^ TIL586 cells in different organs were isolated at 12 days post-injection for subsequent SA-β-Gal staining (C) and ^3^H-thymidine incorporation assays (D). E Intratumoral injection of Poly-G3 enhanced anti-tumor immunity in NSG mice. Human 586mel tumor cells were subcutaneously injected into NSG on day 0. Tumor-specific CD8^+^ TIL586 cells were injected i.v. on day 3 with or without intratumoral injection of Poly-G3. Tumor volumes were measured and presented as mean ± SD (*n* = 5 mice per group). *P*-values were determined by the one-way analysis of variance (ANOVA). Similar results were obtained in three repeat experiments. Data information: Results shown are mean ± SD (*n* = 5 mice per group). **P < 0.01, compared with the respective CD8^+^ TIL586 group recovered from control NSG mice (A, B); ^#^*P* < 0.01, compared with the average levels of CD8^+^ TIL586 group recovered from control NSG mice (A, B); *P < 0.05 and **P < 0.01, compared with the PBS group (C, D) using unpaired *t*-test.

We next investigated whether tumor cells can also convert tumor-specific effector T cells into senescent T cells with suppressive function *in vivo*. Human 586mel tumor cells were subcutaneously injected into NOD-scid *Il2rg*^null^ (NSG) mice to establish 586mel-bearing NSG mice. Human 586mel tumor-specific CD8^+^ TIL586 cells were adoptively transferred into control or 586mel-bearing NSG mice through intravenous injection using the same procedure used in the naïve T-cell studies (Peng *et al*, 2005, 2007). The adoptively transferred CD8^+^ TIL586 cells were recovered at 12 days post-injection, and their senescence and suppressive activity were determined. Importantly, very high percentages of senescent T cells were also induced in transferred CD8^+^ TIL586 cells recovered from different organs and tumor tissues in the tumor-bearing NSG mice. However, purified CD8^+^ TIL586 cells previously transferred into control NSG mice had few senescent T cells (Fig 8A). Furthermore, the CD8^+^ TIL586 cells recovered from different organs and tumor tissues previously transferred into 586mel-bearing mice also had potent suppressive activity and significantly inhibited the proliferation of responding CD4^+^ T cells. In contrast, purified CD8^+^ TIL586 cells previously transferred into control NSG mice did not have suppressive activity (Fig 8B). We further explored whether TLR8 signaling can prevent the induction of senescence and development of suppressive function in tumor-specific CD8^+^ TIL586 cells in this 586mel-bearing NSG mouse model. We used the same experimental procedures as used in the naïve CD4^+^ T-cell studies (Fig 7). We found that intratumoral injection of Poly-G3, but not LPS or PBS, dramatically decreased senescence induction in CD8^+^ TIL586 cells in the 586mel-bearing NSG mice (Fig 8C). In addition, intratumoral injection of Poly-G3 significantly reversed the suppressive function of senescent CD8^+^ TIL586 cells recovered from different organs and tumor sites in the 586mel-bearing mice (Fig 8D). These results further indicate that human tumor cells can also induce tumor-specific effector T-cell senescence with suppressive activity *in vivo* and that TLR8 signaling can prevent these effects on both naïve and effector T cells.

We next determined whether we can enhance anti-tumor immunity by inhibiting tumor-specific T-cell senescence through TLR8 signaling in this adoptive transfer therapy melanoma model. Human 586mel tumor cells were subcutaneously injected into NSG mice. Tumor-specific CD8^+^ TIL586 cells were adoptively transferred through intravenous injection on day 3, followed by intratumoral injection of Poly-G3 or not. As shown in Fig 8E, 586 tumor cells grew progressively in NSG mice. When tumor-specific CD8^+^ TIL586 T cells, which can kill the 586mel, were adoptively transferred, tumor growth was significantly inhibited. Importantly, intratumoral injection of Poly-G3 dramatically promoted the inhibition of tumor growth mediated by tumor-specific TIL586 T cells, suggesting that Poly-G3 treatment enhances the anti-tumor ability mediated by TIL586. We next excluded the possibility that TLR8 ligand Poly-G3 may directly inhibit tumor growth (Cherfils-Vicini *et al*, 2010). We found that TLR8 ligand Poly-G3 did not affect tumor growth *in vivo* in the NSG mice followed by intratumoral injection of Poly-G3 (Supplementary Fig S11). Taken together, our studies clearly indicate that tumor cells can escape anti-tumor immunity by inducing naïve and/or tumor-specific effector T-cell senescence and creating a suppressive tumor microenvironment. In addition, these studies identify a novel strategy for tumor immunotherapy through activation of TLR8 signaling in tumor cells, resulting in enhanced anti-tumor immunity.

## Discussion

Improved understanding of the molecular mechanisms involved in tumor-induced immune suppression and development of effective strategies to reverse tumor suppressive microenvironments are major challenges in the field of clinical tumor immunotherapy. Our current study identified the conversion of naïve/effector T cells into senescent T cells as a novel mechanism utilized by human tumor cells to induce immune tolerance. Our study further demonstrated that tumor-induced T-cell senescence is molecularly mediated by tumor-derived endogenous metabolic cAMP. Most importantly, our results clearly showed that TLR8 signaling can prevent the cAMP production by tumor cells and block tumor-induced conversion of naïve and tumor-specific T cells into senescent cells, resulting in enhanced anti-tumor immunity *in vivo*. These studies identify a novel mechanism related to human tumor-mediated immune suppression and provide an effective strategy to reverse the immunosuppressive effects of tumor microenvironments for tumor immunotherapy.

Accumulation of senescent CD8^+^CD28^null^ T cells has been found in patients with chronic viral infections and with certain types of cancers, but the mechanisms responsible for the induction of these senescent T cells have been unclear (Chen *et al*, 2009; Effros *et al*, 2005; Meloni *et al*, 2006; Tsukishiro *et al*, 2003; Weng *et al*, 2009; Wolfram *et al*, 2000). Our current studies suggested that different types of human cancer cells can induce naïve/effector T-cell senescence. In addition, our more recent studies have demonstrated that human Treg cells can also induce senescence in responder T cells (Ye *et al*, 2012, 2013). These novel findings that both human tumor cells and tumor-infiltrating Treg cells can induce T-cell senescence might at least partially explain the accumulation of senescent T cells in certain patients with cancer. Our studies suggest that induction of T-cell senescence may be a significant mechanism for immune tolerance induction within the tumor suppressive microenvironment. It is now clear that senescent cells have permanent cell cycle arrest but remain viable, metabolically active, and possess a unique transcriptional profile and gene regulation signature (Campisi & d'Adda di Fagagna, 2007). It has strongly suggested that senescent CD8^+^ T cells are functionally active and not anergic lymphocytes (Vallejo, 2005). Senescent CD8^+^ T cells show functional changes and have defective killing abilities due to the loss of perforin and granzyme or defects in granule exocytosis (Appay *et al*, 2000; Vallejo, 2005). In addition, senescent CD8^+^ T cells have negative regulatory functions that reduce the effects of immunization and vaccinations, as well as prolong the survival of allografts (Ciubotariu *et al*, 2001; Cortesini *et al*, 2001; Goronzy *et al*, 2001). In the current study, we showed that tumor-infiltrating T cells from breast cancers, head and neck cancers, and melanomas contained high percentages of senescent T-cell populations and that multiple types of tumor cells can directly induce T-cell senescence. We further showed that these senescent T cells significantly down-regulated expression of co-stimulatory molecule CD28, suggesting dysfunction of these tumor-infiltrating T cells. Importantly, these senescent T cells possessed a potent suppressive activity, strongly suggesting that senescent T cells could indirectly amplify and maintain the immunosuppressive effects mediated by tumor cells and Treg cells in the tumor microenvironment (Ye *et al*, 2012, 2013). In support of this notion, our *in vivo* adoptive transfer studies showed that tumor-bearing microenvironments induced both adoptively transferred human naïve T cells and tumor-specific effector T cells to become senescent T cells possessing suppressive function. These results suggest a potential mechanism for the failures seen in multiple clinical trials of tumor vaccines and adoptive T-cell therapies. In addition, the possibility of blocking the induction of T-cell senescence and restoring the effector function of senescent T cells are critical goals for enhancing anti-tumor immunity.

Tumor cells can utilize multiple strategies to create an immunosuppressive micromilieu and escape the host immune system (Croci *et al*, 2007; Whiteside, 2008). Besides direct expansion and recruitment of various types of immunosuppressive immune cells, such as Treg cells and MSCs (Kiniwa *et al*, 2007; Kusmartsev & Gabrilovich, 2006; Peng *et al*, 2007; Roncarolo *et al*, 2006; Wei *et al*, 2005), our studies showed that tumor cells can also directly induce a conversion from tumor-infiltrating naïve/effector T cells into senescent T cells with potent suppressive activity. Thus, an improved understanding of the molecular mechanisms responsible for the generation of these senescent T cells and their functional alterations in tumor microenvironments should open new avenues for cancer immunotherapy. Increasing evidence suggests that metabolic dysregulation of tumor cells is another key factor involved in tumor-induced suppression. Tumor cells can over-produce several metabolites, such as lactic acid, activated glucose transports and IDO, that contribute to the aggravated immunosuppression in tumor sites (Croci *et al*, 2007). In our efforts to identify the molecules responsible for the induction of T-cell senescence mediated by different types of tumor cells, we found that tumor-derived endogenous cAMP is responsible for the induction of senescence in T cells. There is sufficient evidence showing that cAMP is a critical component of the tumor-induced hypoxic microenvironment and a potent inhibitor of effective tumor-specific T cells (Ohta *et al*, 2006; Sitkovsky *et al*, 2008; Vang *et al*, 2001). Our current studies involving cAMP-mediated T-cell senescence not only identify mechanistic links between tumor immunosuppression, hypoxia, and metabolic dysregulation, but also should lead to novel strategies capable of augmenting immune responses directly against cancer. Notably, our studies also demonstrated that tumor cells can transfer cAMP to targeted T cells via gap junctions, significantly increasing cAMP levels in senescent T cells. Given that human Treg cells can also induce responder T-cell senescence (Ye *et al*, 2012), and cAMP is a key component of Treg cell suppression (Bopp *et al*, 2007), these studies further suggest that senescent T cells induced by tumor cells and tumor-infiltrating Treg cells will induce more senescent T cells, thereby amplifying immunosuppression at tumor sites. We will continue our efforts to identify the molecular mechanisms involved in the regulation of cAMP in different types of tumor cells and in the differentiation and development of tumor-induced senescent T cells.

Recent development of TLR agonists as vaccine adjuvants or therapeutic agents for prevention or treatment of cancer and infectious diseases has become a very active research field. Several TLR ligands, including imiquimod (TLR7) and CpG (TLR9), have shown significant promise for the treatment of cancer (Kanzler *et al*, 2007; Smits *et al*, 2008; Wang *et al*, 2008). These TLR ligands can directly induce TLR-positive tumor cell apoptosis, or enhance tumor-infiltrating innate and tumor-specific T-cell function. They can also reverse immunosuppression by creating a tumor microenvironment favorable for effective anti-tumor immune responses. In contrast, several recent studies have shown that some TLR signaling pathways could promote cancer initiation and progression (Cherfils-Vicini *et al*, 2010; Huang *et al*, 2005). These conflicting results further suggest that TLR signaling regulated in tumor and immune cells is complex and may vary by tumor cell types and different TLR ligands. A better understanding of the function and regulation of TLR signaling pathways in tumor cells and tumor-infiltrating immune cells will not only facilitate the identification of the molecular mechanisms involved in cancer immunopathogenesis, but also will provide novel strategies for the development of effective cancer treatments.

Our previous and current studies strongly indicate the advantages of the development of TLR8-targeted immunity for anti-tumor immunotherapy. First, we recently demonstrated that human TLR8 signaling completely reversed the suppressive functions of naturally occurring CD4^+^CD25^+^ Treg cells and tumor-derived CD4^+^, CD8^+^, and γδ Treg cells (Kiniwa *et al*, 2007; Peng *et al*, 2005, 2007). Furthermore, the TLR8-mediated reversal of Treg suppression can significantly induce enhanced anti-tumor immune responses mediated by tumor-specific CD8^+^ T cells in adoptive transfer tumor models (Peng *et al*, 2005, 2007). Second, our studies suggest that human TLR8 signaling only functionally inactivates Treg cells without changing the Treg repertoire or inhibiting effector T-cell functions. Recent several strategies, such as depletion or blockage of Treg suppressive activities through targeting of CD25, CTLA-4, or PD-1 molecules, have been utilized in animal models and clinical trials and have yielded promising results (Kavanagh *et al*, 2008; Powell *et al*, 2007b). However, these strategies can concurrently eliminate activated effector T cells, prevent effector T-cell activities, and induce Treg cell replenishment, which attenuate the effects of effector T cells on anti-tumor immunity (Colombo & Piconese, 2007; Powell *et al*, 2007a, 2007b; Quezada *et al*, 2006). Third, our current studies further show that human TLR8 signaling can also directly target multiple types of tumor cells and prevent their ability to induce T-cell senescence. The mechanism regulated by TLR8 signaling appears to involve modulation of the levels of the endogenous secondary messenger cAMP in tumor cells, strongly implicating the involvement of metabolic regulation mediated by TLR8 signaling. Finally, in addition to regulating Treg and tumor cells, we more recently observed that TLR8 signaling can also reverse the suppression of senescent T cells induced by Treg and tumor cells and up-regulate co-stimulatory molecules in senescent T cells, resulting in their rejuvenation into effector T cells (Peng, unpublished). Collectively, our studies clearly indicate that human TLR8 signaling can reverse the suppressive effects mediated by tumor microenvironments and switch it into an effector microenvironment, by targeting different types of cells at different levels. Our studies also strongly support the feasibility of the use of TLR8 ligands as tumor immunotherapeutic agents and/or as adjuvants for tumor vaccines. In addition, our novel concept was supported by studies from other groups showing anti-tumor activity mediated by TLR7 and TLR8 ligands (Smits *et al*, 2008; Wang *et al*, 2010). Notably, we did not observe any direct effects on tumor growth in multiple types of tumor cells mediated by TLR8 ligand Poly-G3 *in vitro* and *in vivo*, unlike the results seen with other TLR ligands, such as Poly (I:C) and CpG (Liang *et al*, 2010; Salaun *et al*, 2007). Our future studies will focus on providing a better understanding of the molecular mechanisms and unique signaling pathways involved in the effects of how TLR8 signaling regulates tumor and Treg cell functions. These studies will be critical preludes for the application of TLR8 ligands in tumor therapeutic interventions.

In summary, we identified a novel mechanism mediated by human tumor cells to induce immune suppression that involves conversion of naïve/effector T cells into senescent cells possessing potent suppressive activity. We demonstrated that tumor-derived endogenous cAMP was responsible for the induction of T-cell senescence. Importantly, we discovered that TLR8 signaling in tumor cells can block the induction of senescence in naïve and tumor-specific effector T cells and reverse their suppressive effects *in vitro* and *in vivo*, resulting in enhanced anti-tumor immunity. These studies provide new insights relevant for the development of strategies to prevent or overcome tumor-induced immune suppression.

## Materials and Methods

### Human samples and cell lines

Tumor samples and tumor paired normal tissues of melanoma, breast, ovarian, and colon cancers were obtained from hospitalized patients in the Department of Surgery at St. Louis University from 2004 to 2012 who have given informed consents for enrollment in a prospective tumor procurement protocol approved by the Saint Louis University Institutional Review Board. Buffy coats from healthy donors were obtained from the Gulf Coast Regional Blood Center at Houston. Peripheral blood mononuclear cells (PBMCs) were purified from buffy coats using Ficoll-Paque. Human naïve CD4^+^ and CD8^+^ T cells were purified by EasySep enrichment kits (StemCell Technologies). Different types of tumor cell lines (melanoma, breast, ovarian, prostate and colon cancers, and squamous carcinoma), normal breast tissue-derived cells (BN), were either purchased from the American Type Culture Collection (ATCC, Manassas, VA) or established in our laboratory. Melanoma 586mel and paired TIL586 were obtained from the Surgery Branch, NCI. Breast carcinoma (BC) and colon cancer (CC) cell lines were maintained in keratinocyte medium containing 25 mg/ml bovine pituitary extract, 5 ng/ml epidermal growth factor, 2 mM L-glutamine, 10 mM HEPES buffer, 2% heat-inactivated fetal calf serum (FCS) and penicillin–streptomycin (Invitrogen, Inc.). Other tumor cell lines were maintained in RPMI 1640 medium containing 10% FCS. Hybridoma HB55 and HB95 were obtained from the ATCC.

### Generation of tumor-infiltrating lymphocytes

Tumor and normal tissue-infiltrating lymphocytes were generated from different tumor and normal tissues, as previously described (Peng *et al*, 2007; Su *et al*, 2010). Briefly, tissues were minced into small pieces followed by digestion with collagenase type IV, hyaluronidase, and deoxyribonuclease. After digestion, cells were washed in RPMI1640 and then cultured in RPMI1640 containing 10% human serum supplemented with L-glutamine, 2-mercaptethanol, and 50 U/ml of IL-2 for the generation of T cells.

### Senescence-associated β-Galactosidase (SA-β-Gal) staining

Senescence-associated β-Galactosidase (SA-β-Gal) activity in senescent T cells was detected as we previously described (Ye *et al*, 2012, 2013). Briefly, anti-CD3 activated naïve CD4^+^ or CD8^+^ T cells were co-cultured with or without tumor cells or normal tissue-derived cells at ratio of 1:1 for 1 day and then separated and cultured for additional 3 or 5 days. The treated T cells were washed in PBS (pH 7.2), fixed in 3% formaldehyde, and followed to incubate overnight at 37°C with freshly prepared SA-β-Gal staining solution (1 mg/ml X-Gal, 5 mM K_3_Fe[CN]_6_, 5 mM K_4_Fe[CN]_6_, 2 mM MgCl_2_ in PBS at pH 6.0). The stained cells were washed with H_2_O and examined with a microscope.

For some experiments, SA-β-Gal-positive populations were determined in the co-cultured naïve T cells in the presence of following TLR ligands, neutralizing antibodies, or various inhibitors. TLR ligands included: Pam3CSK4 (200 ng/ml), Poly (I:C) (25 μg/ml), LPS (100 ng/ml), Flagellin (10 μg/ml), Loxoribine (500 μM), ssRNA40/LyoVec (3 μg/ml) (Invivogen, San Diego, CA), and oligonucleotides CpG-B (3 μg/ml), Poly-T3 (3 μg/ml) and Poly-G3 (3 μg/ml) (synthesized by Invitrogen, Carlsbad, CA); neutralizing antibodies included: anti-IL-10 (30 μg/ml) (Clone JES3-19F1, BD Biosciences) and antihuman LAP (TGF- β1 10 μg/ml), anti-TCRαβ 10 μg/ml), anti-PDL-1 (10 μg/ml) (R & D Systems, Minneapolis, MN), anti-MHC-class II (Hybridoma supernatants, 200 μg/ml); ATM inhibitor KU55933 (20 μM, Tocris Bioscience); cAMP and PKA inhibitors included: 2′,5′-Dideoxyadenosine (7ddA, 320 μM) and Dihydrochloride (H89, 20 μM) (Calbiochemistry, San Diego, CA). For forskolin and 3-isobutyl-1-methylxanthine (IBMX) treatment experiments, MCF7, M628, or PC3 tumor cells were pretreated with forskolin (50 μM, Calbiochemistry) or IBMX (200 μM, Sigma) for 2 days, then co-cultured with anti-CD3-activated CD4^+^ T cells and detected for SA-β-Gal expression as described above. In some experiments, anti-CD3-activated CD4^+^ T cells were directly cultured in the presence of forskolin (0, 0.5, 1, 2 μM) for 5 days and then detected for SA-β-Gal expression. For MAPK and NF-κB blockage experiments, tumor cells were pretreated with MAPK inhibitors U0126 (10 μM), SB203580 (10 μM) and SP600125 (10 μM) or NF-κB inhibitor APDC (10 μM) (Calbiochemistry) for 3 days and added with or without Poly-G3 on day 3. The pretreated tumor cells were co-cultured with anti-CD3-activated CD4^+^ T cells at ratio of 1:1 for 1 day. The treated T cells were separated and then detected for SA-β-Gal expression. For CREB siRNA knockdown experiment, anti-CD3-activated CD4^+^ T cells were transfected with the CREB siRNA or nonspecific control siRNA at a final concentration of 100 nmol/L (NM_004379, Sigma). siRNA-transfected CD4^+^ T cells were co-cultured with tumor cells and then purified for SA-β-Gal staining.

### Flow cytometry analysis

The expression markers on CD4^+^ T cells were determined by FACS analysis after surface staining or intracellular staining with antihuman specific antibodies conjugated with either PE or FITC. These human antibodies included: anti-CD4, anti-CD8, anti-CD28, and anti-phosphorylated ATM, which were purchased from BD Biosciences. All stained cells were analyzed on a FACSCalibur flow cytometer (BD Bioscience) and data analyzed with FlowJo software (Tree Star).

### Western-blotting analysis

Anti-CD3 activated naïve CD4^+^ T cells were co-cultured with MCF7 or PC3 for 0, 1, 3, or 5 days. Co-cultured CD4^+^ T cells were purified and then lysates prepared for Western blot analyses. The antibodies used in Western blotting are as follows: anti-phospho-LCK, anti-LCK, anti-phospho-PKA, anti-PKA, anti-phospho-CREB, anti-CREB, anti-actin (Cell Signaling Technology, Danvers, MA) and anti-p53, anti-p21 (C-19), and anti-p16 (Santa Cruz Biotechnology). In some experiments, Poly-G3 was included in the co-cultures and the treated naïve CD4^+^ T cells were then used for Western blot analyses.

### Functional proliferation assay

Proliferation assays were performed as previously described (Peng *et al*, 2005, 2007). In brief, 1 × 10^5^ naïve CD4^+^ T cells freshly purified from healthy donors were co-cultured with tumor or normal tissue cell-treated T cells at different ratios (1:0, 1:0.1, 1:0.2, 1:0.5, and 0:1) in anti-CD3-coated (2 μg/ml) 96-well plates in T-cell assay medium containing 2% human AB serum. After 56 h of culture, [^3^H]-thymidine was added at a final concentration of 1 μCi/well, followed by an additional 16 h of culture. The incorporation of [^3^H]-thymidine was measured with a liquid scintillation counter.

### Calcein AM transfer and Gap junction blockage

MCF7, M628, or PC3 tumor cells were incubated with Calcein AM (5 μM, Invitrogen, Inc.) in PBS for 40 min (37°C, 5% CO_2_). The cells were washed with 5% FBS in PBS twice and co-cultured with anti-CD3-activated CD4^+^ T cells at ratio of 1:1 in the presence or absence of GAP27 (300 μM, Tocris Bioscience) for 1 to 3 days. Calcein AM transfer in T cells was determined by FACS analyses. For gap junction blockage assay, MCF7, M628, or PC3 tumor cells were co-cultured with anti-CD3-activated CD4^+^ T cells in the presence or absence of GAP27 (300 μM) for 1 day. The treated T cells were separated and cultured for an additional 3 days and then studied for SA-β-Gal expression and cAMP concentrations.

### cAMP determination

Tumor cells or T cells were washed three times in ice-cold PBS and then lysed by freezing and thawing for three times. The concentrations of cytosolic cAMP in cell lysates were measured by a cAMP-specific ELISA according to the manufacturer's instructions (R & D Systems).

### Lentivirus-shRNA generation and gene knockdown in tumor cells

The methods for design and construction of shRNA specific for TLR8, IRAK4, MyD88, ERK1, ERK2, P38α, JNK1 and IKKα or scramble lenti-shRNAs, and generation of recombinant lentivirus carrying GFP and shRNA, have been described previously (Peng *et al*, 2005, 2007). For virus transfection, concentrated lentiviral supernatant with a multiplicity of infection (MOI) of 5-10 in a total volume of 0.5 ml culture medium was added to the tumor cells growing in 24-well plates containing 8 μg/ml polybrene (Sigma) and then centrifuged at 1000 × g for 1 h at room temperature. Tumor cells were sorted into GFP^+^ and GFP^−^ cells with a FACS ARIA sorter at 3 or 4 days post-transfection. The sorted cells (GFP^+^ and GFP^−^) were then used to determine their capacity for T-cell senescence induction and cAMP production.

### Reverse-transcription–PCR analysis

Total RNA was extracted from tumor cells using Trizol reagent (Invitrogen), and cDNA was transcribed using a SuperScript II RT kit (Invitrogen), both according to manufacturers' instructions. TLR1 to TLR9 mRNA expression were determined by reverse-transcription–PCR using specific primers, and mRNA levels in each samples were normalized to the relative quantity of Glyceraldehyde-3-phosphate dehydrogenase (GAPDH) as previously described (Peng *et al*, 2005, 2007).

### *In vivo* studies

*Rag1*^−/−^ mice and NOD-scid *Il2rg*^null^ mice (NSG, lacking T and B cells) were purchased from The Jackson Laboratory and maintained in the institutional animal facility. All animal studies have been approved by the Institutional Animal Care Committee. Human 586mel tumor cells (5 × 10^6^) in 100 μl of buffered saline were subcutaneously injected into *Rag1*^−/−^ mice or NSG mice. Anti-CD3 (2 μg/ml) preactivated naïve CD4^+^ T cells (5 × 10^6^/mouse), or tumor-specific CD8^+^ TIL586 cells (5 × 10^6^), which recognized and kill 586 mel cells, were adoptively transferred into control *Rag1*^−/−^ or tumor-bearing mice (tumor size of 10 × 10 mm) through intravenous injection. In a parallel experiment, PBS (100 μl/mouse), LPS (10 μg, 20 μg or 50 μg in 100 μl PBS/mouse), Poly-G3 (50 μg in 100 μl PBS/mouse), or 7-ddA and H89 (0.64 nmol and 0.05 nmol in 100 μl PBS/mouse, respectively) were injected into tumors 1, 4, 7, or 10 days after adoptive transfer of CD4^+^ T cells into the mice. Five to ten mice were included in each group. Blood, lymph nodes (LN), spleens (SP), and tumors were harvested at 12 days post-injection; and mononuclear cells were purified over Ficoll. The transferred human CD4^+^ T cells were isolated and recovered by antibody-coated microbeads for subsequent phenotypic and functional analyses *in vitro*. SA-β-Gal staining, cAMP detection, and ^3^H-thymidine incorporation assays were performed as previously described.

**The paper explained**

**Problem**

Immunotherapy is a promising approach for treating patients with advanced cancer. However, an immunosuppressive microenvironment created by malignant tumors represents a major obstacle for effective anti-tumor immunity. A better understanding of the suppressive mechanisms mediated by the tumor microenvironment and the development of strategies to reverse the immune suppression are major challenges for the success of tumor immunotherapy.

**Results**

We discover that human tumor cells can induce senescence in naïve/effector T cells, exhibiting potent suppressive function *in vitro* and *in vivo*. We further show that tumor-derived endogenous cyclic adenosine monophosphate (cAMP) is responsible for the induction of T-cell senescence. Importantly, activation of TLR8 signaling in tumor cells can block the induction and reverse the suppression of senescent naïve and tumor-specific T cells *in vitro* and *in vivo*, resulting in enhanced anti-tumor immunity.

**Impact**

This manuscript identifies a novel mechanism of human tumor-mediated immune suppression and provides a new strategy using TLR8 ligands to reverse tumor immunosuppressive effects for tumor immunotherapy.

For tumor growth and anti-tumor immunity studies, human 586mel tumor cells (5 × 10^6^) in 100 μl of buffered saline were subcutaneously injected into NSG mice. Tumor-specific CD8^+^ TIL586 cells (5 × 10^6^) were i.v. injected on day 3 with or without intratumoral injection of Poly-G3 for a total of 4 doses (3 days interval). Tumor size was measured with calipers every 4 days. Tumor volume was calculated on the basis of two-dimension measurements.

### Statistical analysis

Statistical analysis was performed with GraphPad Prism5 software. Unless indicated otherwise, data are expressed as mean ± standard deviation (SD). D'Agostino and Pearson test was used to test whether the data come from a Gaussian distribution. For multiple group comparison *in vivo* studies, the one-way analysis of variance (ANOVA) was used, followed by the Dunnett's test for comparing experimental groups against a single control. For single comparison between two groups, paired Student's *t*-test was used. Nonparametric *t*-test was chosen if the sample size was too small and not fit Gaussian distribution.
