# Supplementary material for: TLR8 signaling enhances tumor immunity by preventing tumor-induced T-cell senescence
Source: EMBO Mol Med. 2014 Sep 17;6(10):1294–311. doi: 10.15252/emmm.201403918 (PMC4287933; doi:10.15252/emmm.201403918)

Supplemental figure legends

Figure S1

Tumor cells induce T cell senescence in co-cultures.

(A) High populations of senescent $CD3^+$ T cells existed in TILs compared with those in normal breast tissue-derived lymphocytes. $CD3^+$ T cells were directly purified from the freshly digested tumor or normal tissues by microbeads and SA-β-gal staining was determined. BN: Normal breast tissues; MC: melanoma tissues; BC: breast cancer tissues; and CC: colon cancer tissues. **(B)** MCF7 and PC3 treatment significantly increased SA-β-Gal positive T cell populations in co-cultured naïve $CD4^+$ T cells. Anti-CD3 activated naïve $CD4^+$ T cells were co-cultured with tumor cell lines or normal breast cells at ratio of 1:1 for 1 day. The treated $CD4^+$ T cells were then separated and stained with SA-β-Gal staining reagents after culture for 3 additional days. The SA-β-Gal positive T cells were identified with dark blue granules as the arrows indicate. **(C)** Primary tumor cells derived from fresh melanoma, breast cancer colon cancer tumor tissues also induced T cell senescence. Cell treatment and procedure were the same as in (A). SA-β-Gal expression was measured in the treated $CD4^+$ T cells.

Figure S2

Cell-cell contact is required for tumor-induced T cell senescence.

(A) Culture supernatants from tumor cell lines did not promote the induction of SA-β-Gal positive T cell populations in naïve $CD4^+$ T cells. Anti-CD3-activated naïve $CD4^+$ T cells were either co-cultured with tumor cells as described in Figure 1, or cultured with different concentrations of culture supernatants collected from tumor cell lines for three days. SA-β-Gal expression of the treated $CD4^+$ T cells was then determined. **(B)** The loss of CD28 expression in tumor-treated T cells cannot be prevented through the blockage of IL-10, TGF-β, TCRαβ, PDL-1, MHC-class II, and IDO pathways. Tumor cells and naïve $CD4^+$ T cells were pretreated with the indicated neutralizing antibodies or the IDO inhibitor 1-MT, and then co-cultured in the presence or absence of these neutralizing antibodies and 1-MT. The co-cultured $CD4^+$ T cells were then separated and analyzed for CD28 expression by flow cytometry.

Figure S3

Tumor cells are able to transfer calcein AM through gap junctions to the co-cultured T cells.

(A) Significantly increased calcein AM positive T cell populations were detected after co-culture with calcein AM-labeled tumor cells. **(B)** Gap junction inhibitory peptide GAP27 markedly decreased the calcein AM positive T cell populations in naïve $CD4^+$ T cells co-cultured with calcein AM-labeled tumor cells. Anti-CD3 activated naïve $CD4^+$ T cells were co-cultured with calcein AM-labeled MCF7, M628 and PC3 tumor cells in the presence or absence of GAP27 (300 μM) for 1 to 3 days. Calcein AM transfer in T cells was determined by FACS analyses.

Figure S4

Blockage of cAMP signaling prevents the induction of T cell senescence mediated by tumor cells.

(A) Inhibition of cAMP signaling by specific pharmacological inhibitors significantly reversed T cell senescence induced by tumor cells. Anti-CD3 pre-activated naïve $CD4^+$ T cells were co-cultured with tumor cells in the presence or absence of inhibitors 7-ddA (320 μM), or H89 (20 μM) or the combination for 1 day. Co-cultured $CD4^+$ T cells were separated and SA-β-Gal expression was determined after culture for an additional 3 days. Data are mean of three independent experiments ± SD. *p<0.05 and ** p<0.01, compared with the group not treated with inhibitors using paired t-test. **(B)** Pretreatment of tumor cells with inhibitors 7ddA and H89 significantly decreased SA-β-Gal positive T cell populations in naïve $CD4^+$ T cells induced by tumor cells. MCF7, PC3, or M628 cells were pretreated with 7-ddA (320 μM), H89 (20 μM), or combination for 1 day, and then co-cultured with anti-CD3 activated $CD4^+$ T cells for another 1 day. $CD4^+$ T cells were separated and analyzed for SA-β-Gal expression after culture for 3 additional days. Data are mean of three independent experiments ± SD and paired t-test was performed. *p<0.05 and ** p<0.01, compared with the group not treated with inhibitor.

Figure S5

cAMP is involved in the induction of T cell senescence mediated by tumor cells.

(A) Pretreatment of tumor cells with forskolin or IBMX significantly promoted the induction of T cell senescence in naïve T cells co-cultured with tumor cells. MCF7, M628 or PC3 tumor cells were pretreated with forskolin (50 μM) or IBMX (200 μM) for 2 days, and then co-cultured with anti-CD3-activated $CD4^+$ T cells for 1 more day. The treated T cells were separated and SA-β-Gal expression determined after culture for 3 additional days. **(B)** Forskolin treatment of naïve T cells can directly induce T cell senescence. Anti-CD3-activated $CD4^+$ T cells were cultured in the presence of forskolin (0, 0.5, 1, or 2 μM) for 5 days, and SA-β-Gal expression determined. * $p<0.05$ and ** $p<0.01$, compared with the medium only group. Data shown are as mean of three independent experiments ± SD using paired t-test.

Figure S6

cAMP signaling mediates DNA damage response in T cells.

(A) Inhibition of the cAMP signaling by 7-ddA, H89, or the combination, significantly inhibited the phosphorylation of ATM in tumor-treated T cells. MCF7, M586, and PC3 tumor cells were pretreated with cAMP inhibitors 7ddA (320 μM) and/or H89 (20 μM) and then cocultured with anti-CD3-activated $CD4^+$ T cells for 1 day. The co-cultured $CD4^+$ T cells were separated and the p-ATM expression determined after culture for 3 additional days using FACS analyses. **(B)** Forskolin treatment of naïve T cells can markedly increase the ATM phosphorylation. Anti-CD3 activated $CD4^+$ T cells were treated with forskolin (1 or 2 μM) for 5 days and the p-ATM expression determined by FACS analyses.

Figure S7

cAMP-induced PKA-CREB pathway involves T cell senescence.

(A) Inhibition of the cAMP signaling by 7-ddA and H89 dramatically decreased phosphorylation of CREB in $CD4^+$ T cells co-cultured with tumor cells. MCF7, M586, and PC3 tumor cells were pretreated with cAMP inhibitors 7ddA and/or H89 and then cocultured with anti-CD3-activated $CD4^+$ T cells for 1 day. The co-cultured $CD4^+$ T cells were separated and the p-CREB expression determined after culture for 3 additional days using FACS analyses. **(B)** Knockdown of CREB expression in $CD4^+$ T cells with specific siRNA significantly prevented tumor cell-induced $CD4^+$ T cell senescence. Anti-CD3-activated $CD4^+$ T

cells were transfected with the CREB siRNA or nonspecific control siRNA. siRNA-transfected $CD4^+$ T cells were co-cultured with tumor cells for 1 day, and then purified for the detection of SA-β-Gal expression after culture for 3 additional days.

Figure S8

RT-PCR analysis of TLR mRNA expression in primary tumors and tumor cell lines.

TLR1 to TLR9 mRNA expression were determined by RT-PCR using specific primers, and mRNA levels in each sample were normalized to the relative quantity of GAPDH. CC1 and OC1556 are primary tumor cells derived from fresh colon cancer (CC) and ovarian tumor (OC) tissues.

Figure S9

Knockdown of ERK1/2 and/or p38α in MCF7 and PC3 tumor cells blocks the Poly-G3-mediated reversal of tumor-induced $CD4^+$ T cell senescence.

Tumor cells were transfected with lenti-shRNAs specific for ERK1, ERK2, p38α, JNK, or IKKα molecules. Transduced (GFP^+) tumor cells were purified by FACS sorting and co-cultured with anti-CD3 activated $CD4^+$ T cells in the presence or absence of Poly-G3 for 1 day. The treated $CD4^+$ T cells were then separated and SA-β-Gal expression determined after culture for 3 additional days. *$p<0.05$ and ** $p<0.01$, compared with the group transfected with control shRNA using paired t-test.

Figure S10

Intratumoral injection of 7-ddA and H89 can significantly decrease the cAMP levels in transferred $CD4^+$ T cells in human 586mel-bearing mice.

Cell preparation and injection procedures were the same as in Figure 7. 7-ddA (0.64nmol in 100 μl PBS/mouse) and H89 (0.05nmol in 100 μl PBS/mouse) were injected into tumors on days 1, 4, 7, and 10 after adoptive transfer of $CD4^+$ T cells into $Rag1^{-/-}$ 586mel tumor-bearing mice. The transferred human $CD4^+$ T cells in blood, SP, LN and tumors were isolated at 12 days post injection for subsequent

cytosolic c-AMP detection. Results shown are mean ± SD (n= 5 mice per group) and unpaired t-test was performed. * $p<0.05$ compared with the with PBS group.

Figure S11

TLR8 ligand Poly-G3 did not affect tumor growth *in vivo*.

Human 586mel tumor cells were subcutaneously injected into NSG mice on day 0. LPS (10 μg and/or 20 μg in 100 μl PBS/mouse), or Poly-G3 (50 μg in 100 μl PBS/mouse), or PBS was injected into tumors on days 1, 4, 7, and 10. Tumor volumes were measured and presented as mean ± SD (n = 5 mice per group). Similar results were obtained in three repeat experiments.

Fig. S1 Ye, et al.

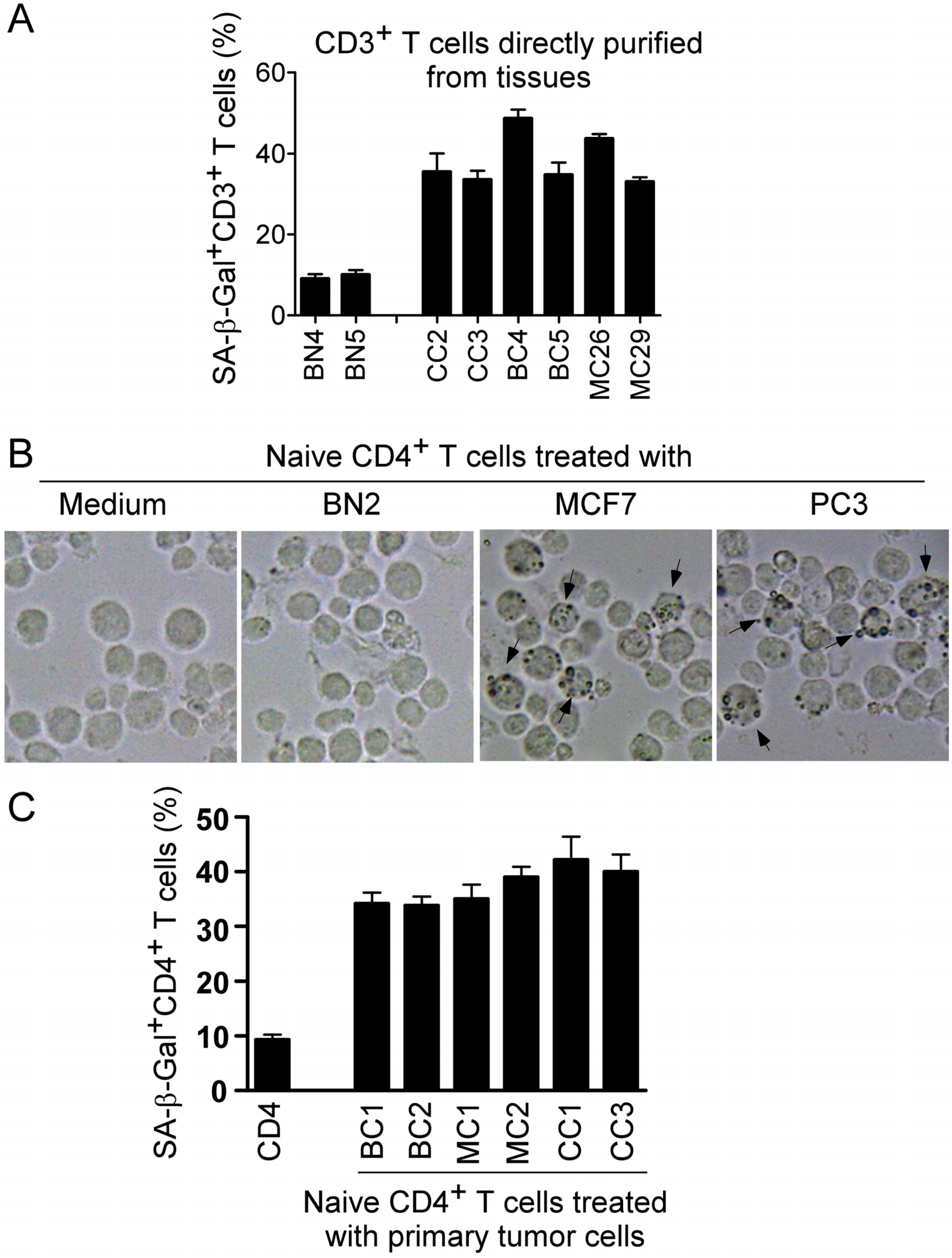

Fig. S2 Ye, et al.

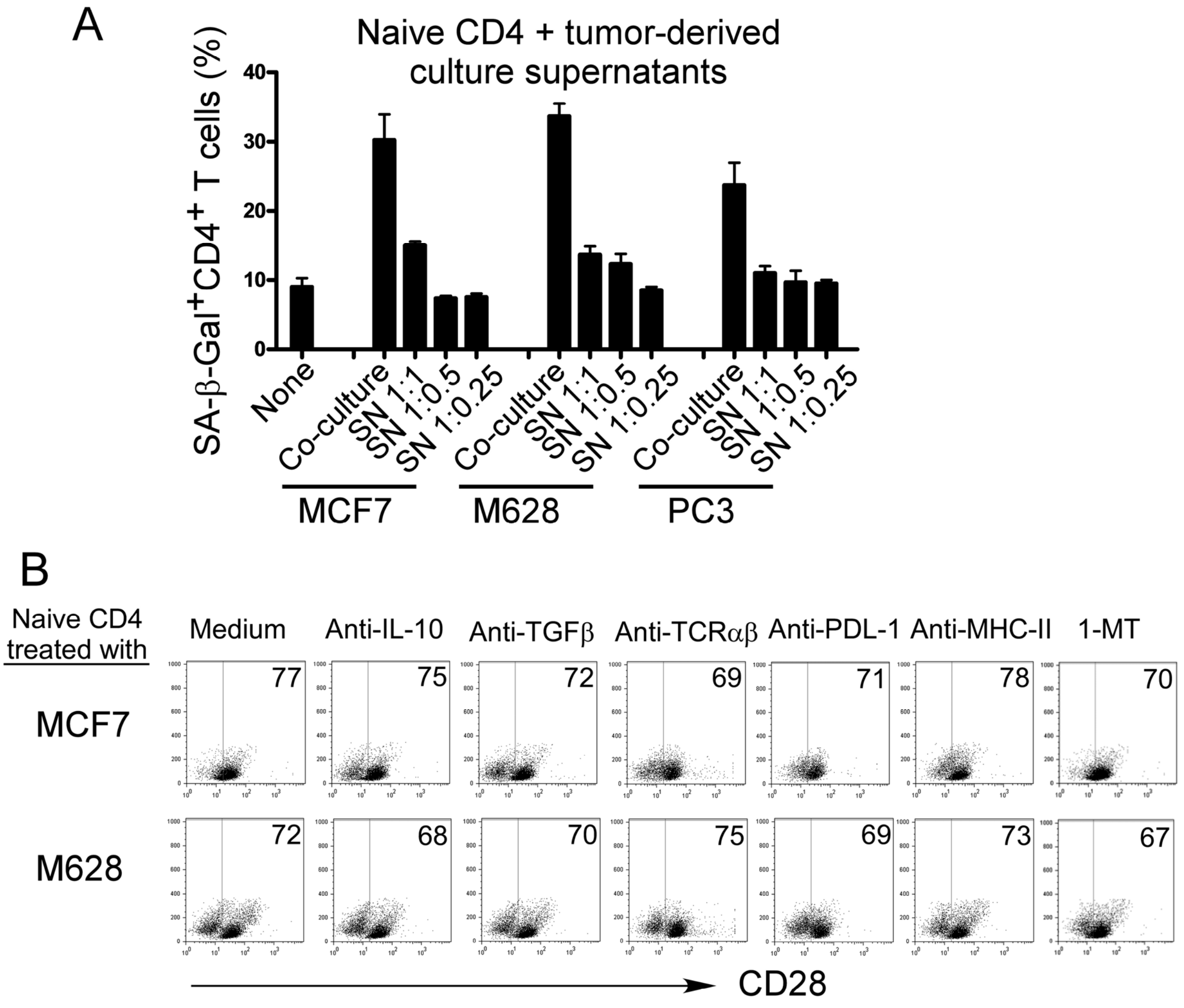

Fig. S3 Ye, et al.

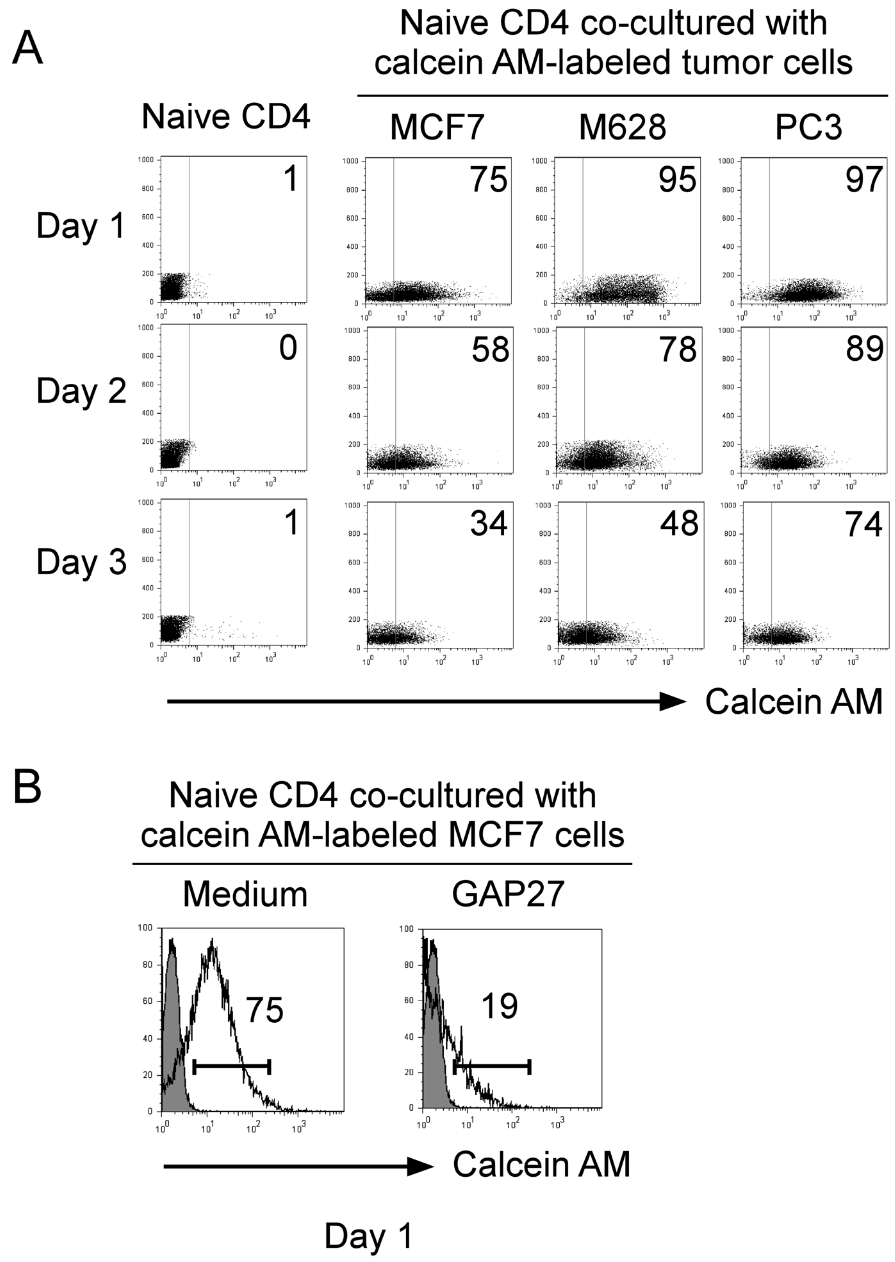

Fig. S4 Ye, et al.

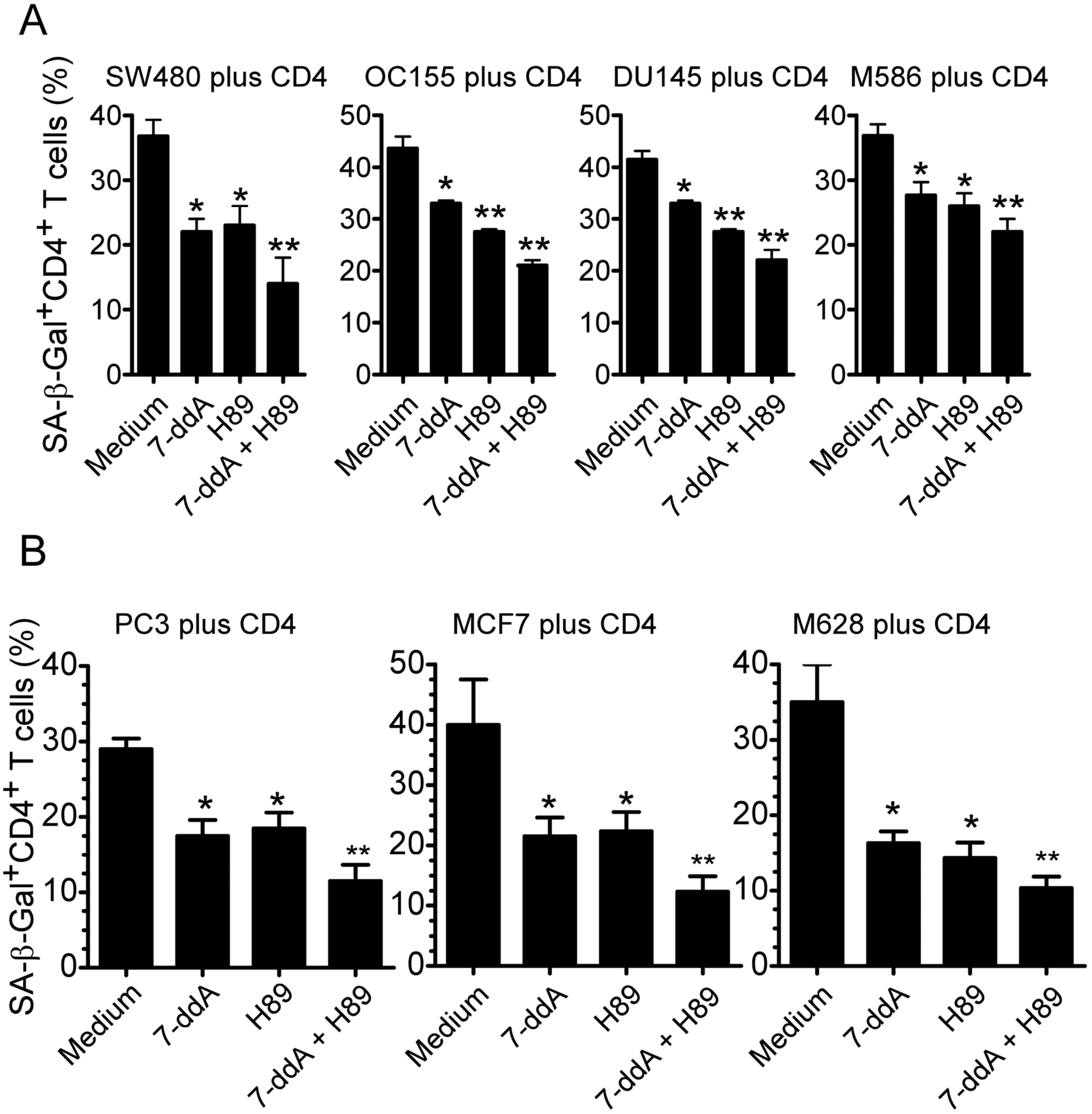

Fig. S5 Ye, et al.

A Tumor cells pretreated with forskolin and IBMX

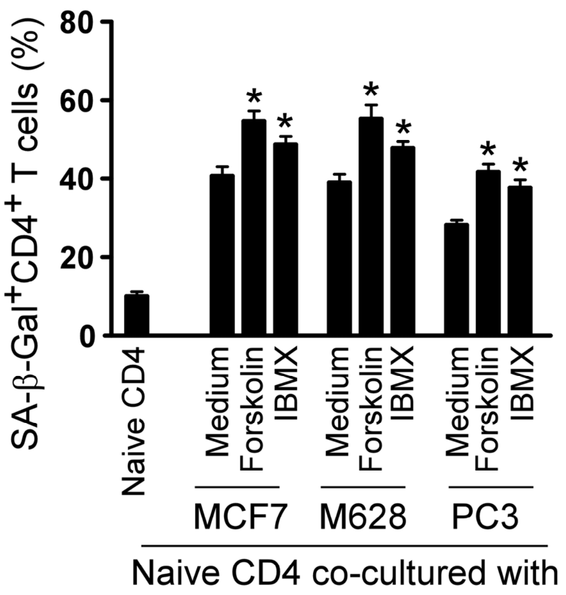

B Naive CD4⁺ T cells treated with forskolin

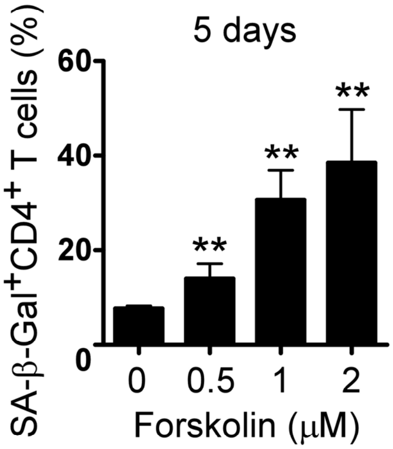

Fig. S6 Ye, et al.

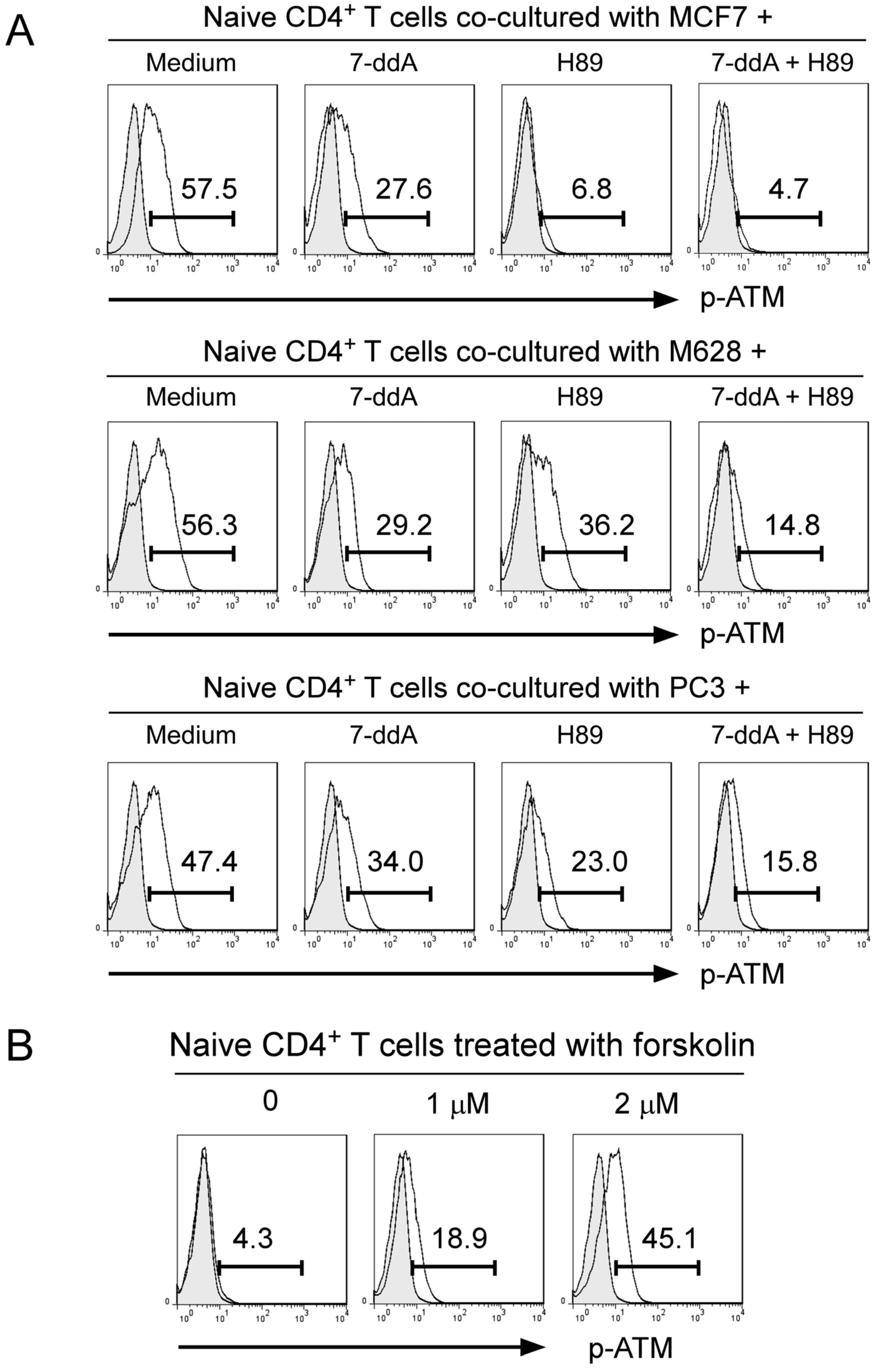

Fig. S7 Ye, et al.

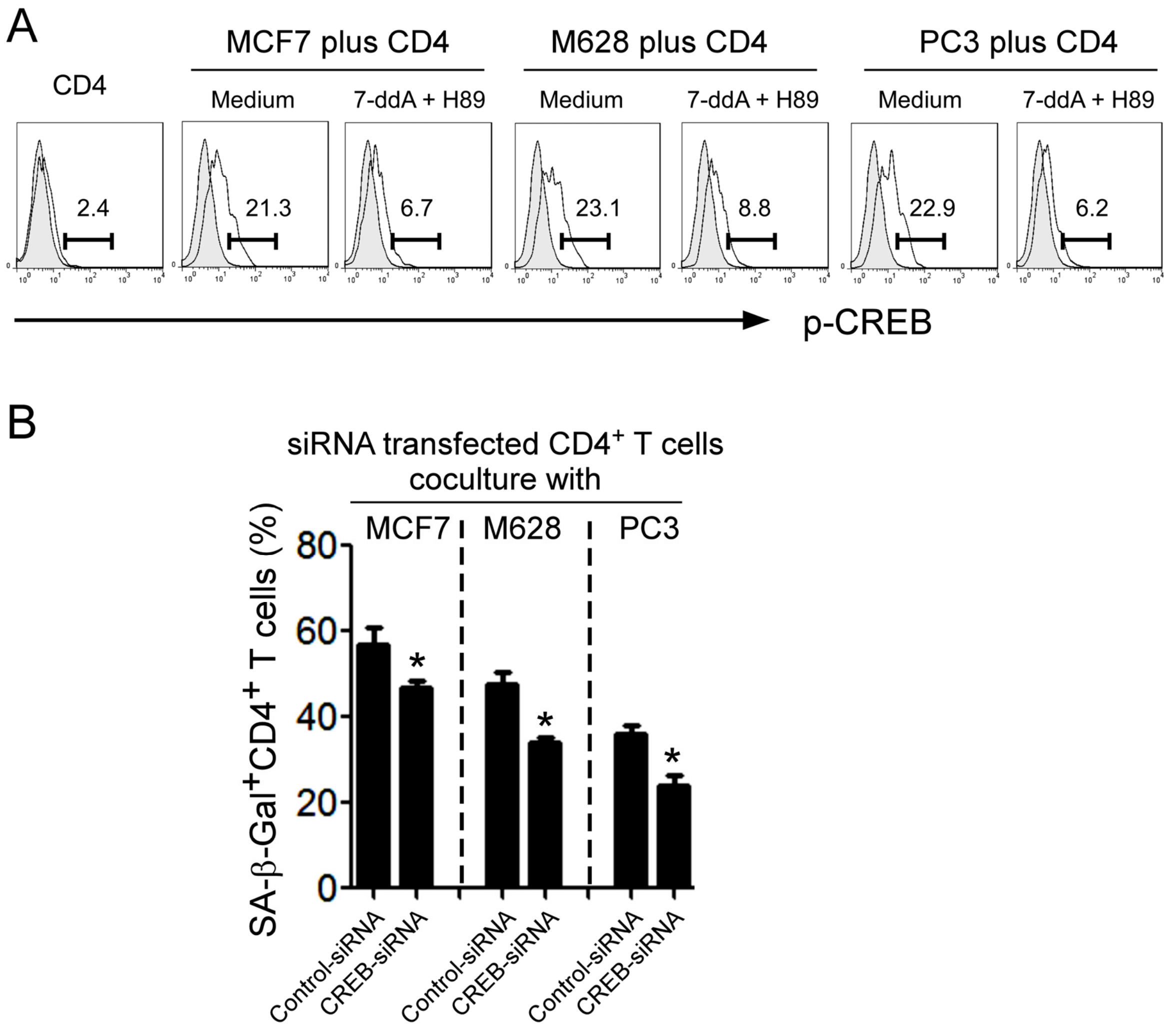

Fig. S8   Ye, et al.

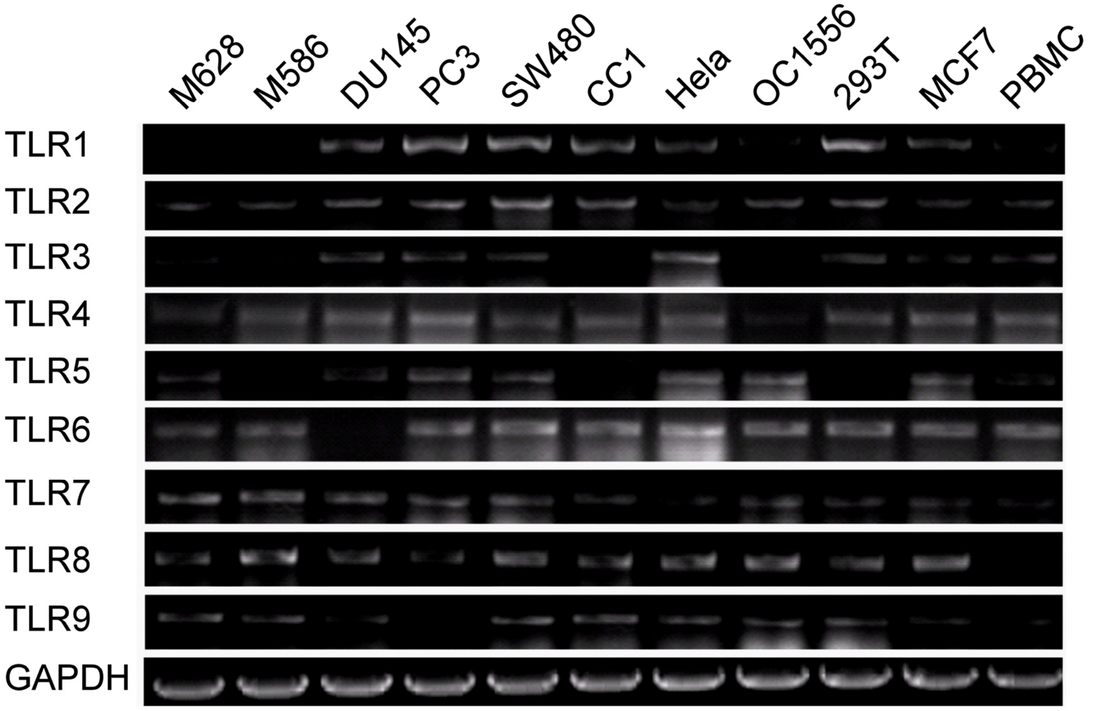

Fig. S9 Ye, et al.

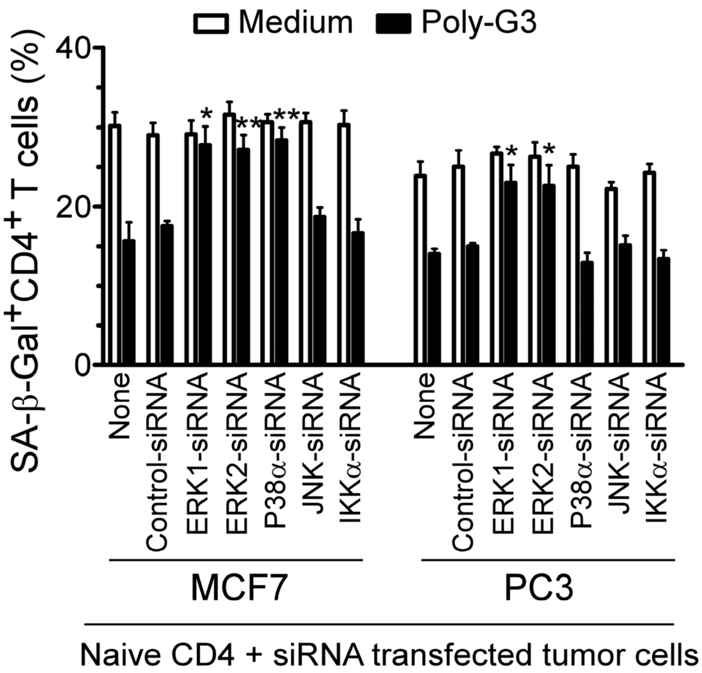

Fig. S10 Ye, et al.

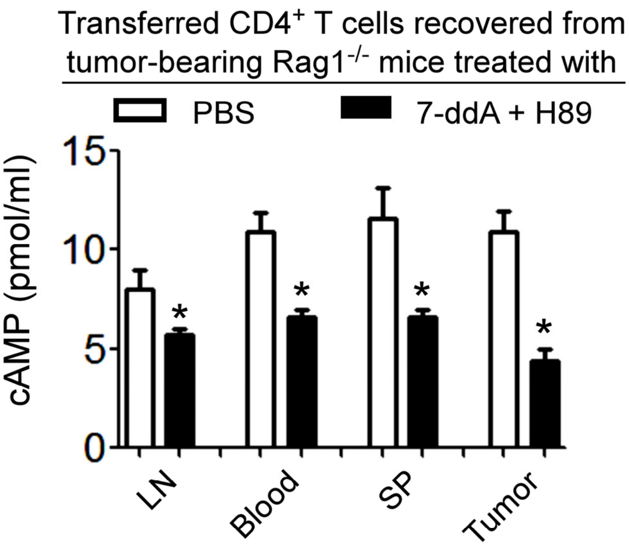

Fig. S11 Ye, et al.

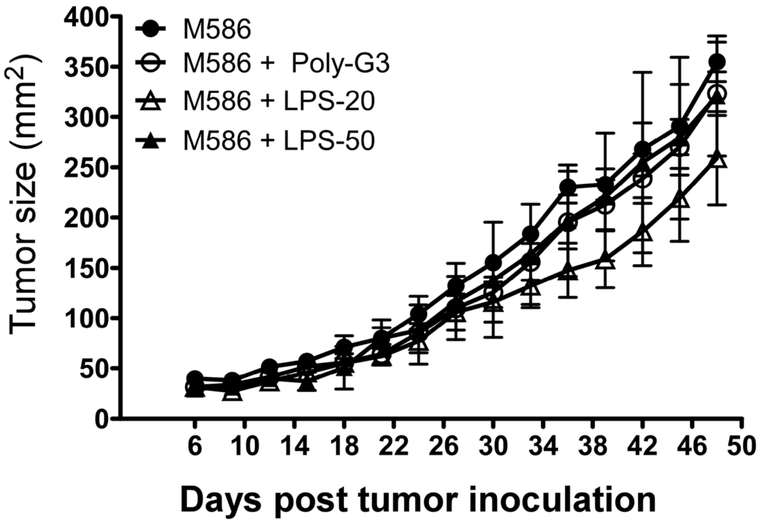

Supplement: Supplementary file 1 [file emmm0006-1294-sd1.pdf]
