# Supplementary material for: TLR8 signaling enhances tumor immunity by preventing tumor-induced T-cell senescence
Source: EMBO Mol Med. 2014 Sep 17;6(10):1294–311. doi: 10.15252/emmm.201403918 (PMC4287933; doi:10.15252/emmm.201403918)
Supplement: Supplementary file 2 [file emmm0006-1294-sd2.pdf]

TLR8 signaling enhances tumor immunity by preventing tumor-induced T cell senescence

Jian Ye, Chunling Ma, Eddy C. Hsueh, Jie Dou, Wei Mo, Shuai Liu, Bing Han, Yi Huang, Yanping Zhang, Mark A. Varvares, Daniel F. Hoft and Guangyong Peng

Corresponding author: Guangyong Peng, Saint Louis University

Review timeline:

Submission date:	29 January 2014
Accepted:	13 August 2014

Editor: Céline Carret

Transaction Report:

No Peer Review Process File is available with this article, as the authors have chosen not to make the review process public in this case.
